# Evolving Dynamics of Whole-Genome Influenza A/H3N2 Viruses Isolated in Cameroon

**DOI:** 10.1155/av/3668615

**Published:** 2025-09-19

**Authors:** Desmon Toutou Tsafack, Chavely Gwladys Monamele, Moïse Henri Moumbeket-Yifomnjou, Loique Landry Messanga Essengue, Chanceline Ndongo Bilounga, Mohamadou Ripa Njankouo, Pascal Ibrahim Touoyem, Ubald Tamoufe, Francioli Koro Koro, Richard Njouom

**Affiliations:** ^1^Virology Department, Centre Pasteur Cameroon, Yaoundé, Cameroon; ^2^Department of Biochemistry, University of Douala, Douala, Cameroon; ^3^Faculty of Health Sciences, University of Buea, Buea, Cameroon; ^4^Department for the Control of Diseases, Epidemics and Pandemics, Ministry of Public Health, Yaounde, Cameroon; ^5^Health and Development in Action (HEADA), Yaounde, Cameroon

**Keywords:** Cameroon, evolving dynamics, influenza, next-generation sequencing, whole-genome

## Abstract

**Background:** Since 2023, Cameroon has recorded numerous cases of seasonal influenza caused by the A/H3N2 subtype, which is the strain most commonly encountered worldwide in 2024.

**Methods:** To describe the evolutionary dynamics of influenza A/H3N2 viruses, whole genome sequencing was performed using the Oxford Nanopore Technologies sequencing platform and the SQK-LSK109, EXP-NBD196 reagent kit (Oxford Nanopore Technologies, catalog no. SQK-LSK109). Subsequently, mutational analysis was performed on the 8 genes of the H3N2 influenza strains isolated between 2023 and 2024 in Cameroon by aligning our protein sequences with the reference sequences recommended by the WHO in the northern hemisphere during the 2023-2024 influenza season using MEGA 11 software. The trimeric and tetrameric structures of the HA, NA, and M proteins were downloaded from the protein website https://www.rcsb.org/ and imported into the PyMOL Version 2.6.1 software for visualization and annotation of the observed amino acid substitutions.

**Results:** All Cameroonian A(H3N2) strains from 2023 to 2024 belonged to clade 3c.2a. The mutations I208F, K156I, E66K, N112S, G69N, V239I, K292E, Q189R, G241D, A202D, T3A, S70R, N161S, N138D, N394S, and N120D were detected in most HA1 gene samples (Supporting Table S1). Among these mutations, the important A202D and N161S mutations in HA1 in 2023 and 2024 led to the virulence of the virus and consequently resulted in the rapid evolution of the A/H3N2 virus and the generation of the new clades 3C.2a1b.2a.2a.3 and 3c.2a1b.2a.2a.3a.1, respectively. Similarly, amino acid substitutions at sites I469T, I65V, and H275Y in the NA protein were observed compared to the 2024 vaccine strain A/Darwin/6/2021. We noted the presence of the H275Y substitution in 30% of Cameroonian strains associated with major resistance to neuraminidase inhibitors, particularly oseltamivir. In general, the number of amino acid mutations observed between circulating strains and the vaccine strain for the following year was higher, indicating that circulating strains would evolve away from vaccine strains for the year 2023-2024.

**Conclusions:** These results highlight the evolutionary nature of the human influenza virus.

## 1. Background

Influenza virus infection poses a serious threat to human life and health. Each year, approximately one billion cases of seasonal influenza are recorded, including 3–5 million severe cases, resulting in 290,000 to 650,000 respiratory-related deaths [1, 2], making it one of the most significant public health issues worldwide. Seasonal H3N2 influenza virus is an 8-segmented RNA virus, encompassing the genes for hemagglutinin (HA), neuraminidase (NA), matrix protein (MP), nonstructural (NS), nucleoprotein (NP), polymerase acidic (PA), polymerase basic 1 (PB1), and polymerase basic 2 (PB2). These genetic segments collectively encode essential proteins including HA, NA, MP 1 (M1), MP 2 (M2), nonstructural protein (NS1), nuclear export protein (NEP), NP, PA, PA-X, PB1, PB1-F2, and PB2 [3, 4]. Based on the major surface antigens, there are 18 HA (H1–H18) and 11 NA (N1–N11) subtypes of the influenza A virus [5]. Since their emergence in 1968, influenza H3N2 viruses have been highly prevalent, with the H3 HA and N2 NA surface glycoproteins being the dominant proteins in these strains [6]. The activities of both HA and NA are crucial for viral function, with antibodies targeting these proteins serving as the primary defense against infection [7]. HA, the main surface antigen of the H3N2 seasonal influenza virus, comprises five antigenic sites: Region A (amino acids [AAs] 138, 142, 144, 145), Region B (AAs 128, 156, 159, 160, 186, 198), Region C (AAs 45, 48, 50, 53, 278, 280, 311, 312), Region D (AAs 171, 212, 214, 219, 230, 246), and Region E (AAs 62, 75, 78, 94, 260, 261) [8]. While the dynamics of influenza epidemics are complex and not fully understood, one major driver of seasonal variation is antigenic drift [9]. For example, in Canada, antigenic drift was observed in 2008 when the A/Brisbane/10/2007 strain mutated into the A/Perth/16/2009 strain, which was isolated for the first time in Australia and grouped within clade 3C.3a and was used as a vaccine strain in subsequent years [10]. In Cameroon, Monamele et al. confirmed several antigenic site mutations among H3N2 virus strains during the 2014–2016 influenza seasons. This study briefly describes the frequency of influenza cases and the evolutionary dynamics of the H3N2 seasonal influenza virus in Cameroon over two successive years, from 2023 to 2024. In addition, it carries out comprehensive analyses of homology, evolution, and variation within the complete genomic sequences of 33 strains of the H3N2 seasonal influenza virus over the same period. These results make a significant contribution to our understanding of the evolutionary and variation characteristics of the complete genome of the H3N2 seasonal influenza virus, providing up-to-date epidemiological data for predicting future influenza epidemics and prevention and control strategies.

## 2. Materials and Methods

### 2.1. Sample Collection and Preparation

In accordance with authorization no. 3971CEI-Udo/07/2023/M from the Ethical and Institutional Committee of the University of Douala, nasopharyngeal swabs were collected from patients with acute respiratory infection (ARI) at 19 influenza sentinel surveillance sites in Cameroon between January 2023 and December 2024. ARI was defined according to the European Centre for Disease Prevention and Control (ECDC) guidelines, which include patients with influenza-like illness (ILI) or severe acute respiratory infection (SARI) according to the WHO case definitions. Nasopharyngeal specimens were collected using polyester swabs and stored at 4°C in 2 mL virus transport medium and transported to the Centre Pasteur of Cameroun (CPC). Samples were processed immediately or stored at −80°C prior to analysis.

### 2.2. Extraction of Viral Nucleic Acids and RT-PCR

Viral RNA was extracted from nasopharyngeal swabs using the QIamp viral RNA Kit (Qiagen, Hilden, Germany) according to the manufacturer's recommendations. In summary, 140 μL of nasopharyngeal swab specimen was utilized for nucleic acid extraction. Detection and typing/subtyping of influenza viruses were performed using the SuperScript III Platinum One-Step Quantitative RT-PCR (qRT-PCR) System (Invitrogen, USA). All samples were tested with a multiplex kit targeting influenza A and B viruses. Positive influenza A samples were further subtyped for A/H3N2. Amplification was conducted on an ABI Prism 7500 thermocycler (Applied Biosystem, Foster City, CA, USA). A 20 μL master mix was prepared, consisting of 1 µL water, 12.5 µL buffer (2X), 0.5 µL enzyme reverse transcriptase/Taq polymerase, 2 µL forward and 2 µL reverse primers (10 μM each), and 2 µL probe (2.5 µM). Five microliters of extracted RNA were added to each sample or control (negative and positive). A threshold cycle (Ct) value below 37 was considered positive. Nucleic acid testing was completed within 24 h.

### 2.3. Whole Genome Sequencing of A (H3N2)

For whole genome sequencing, viral RNA was extracted and subjected to capture and amplification using the ULSEN Ultra-sensitive Influenza Virus Whole Genome Capture Kit (Low Load) from Beijing We Future Technology Co., Ltd. (Catalog no. V-090417). Before the PCR amplification step, superscript III, which is a more thermally stable enzyme than other reverse transcriptases and can operate at higher temperatures (up to 55°C or even 60°C), was first used to convert RNA into cDNA. Next, to fragment the DNA and repair the ends of these fragments, we used the Ultra II enzyme. Finally, the library was prepared by attaching adapters using Quick T4 DNA ligase. The protocol involved incubating the sample at 42°C for 50 min (1 cycle), followed by denaturation at 94°C for 30 s. This was followed by 4 cycles of denaturation at 94°C for 30 s, annealing at 57°C for 30 s, and extension at 68°C for 3 min and 30 s. This was followed by 10 cycles of denaturation at 94°C for 30 s, annealing at 57°C for 30 s, and extension at 68°C for 3 min and 30 s. The process was completed with a single final extension at 68°C for 10 min, followed by cooling to 4°C. The products obtained were purified using the AMPure XP beads nucleic acid purification kit (Beckman Coulter: catalog no. A63880, A63881, A63882). Nucleic acid quantification was performed using the Qubit 4 dsDNA HS Assay Kit fluorometer (Thermo Fisher Scientific: catalog no. Q32851-100 assays). Next, cDNA fragmentation was performed using the NEBNext Ultra II End Repair/dA-Tailing Module Kit from Oxford Nanopore Technologies (USA, catalog no. E7546). Adapter ligation was performed using the NEBNext Quick Ligation Module Kit from New England Biolabs (NEB: catalog no. E6056). Finally, whole genome sequencing was performed using the Oxford Nanopore Technologies sequencing platform and the SQK-LSK109 reagent kit, EXP-NBD196 (Oxford Nanopore Technologies, catalog no. SQK-LSK109).

### 2.4. Sequence Alignment and Analysis

The sequencing data were processed and analyzed using BioEdit (Version 7.2.5). The HA, NA, and M sequences of the northern hemisphere influenza virus reference strains for 2023–2024 (A/Darwin/6/2021) used in the phylogenetic analysis were obtained from the Global Initiative on Sharing All Influenza Data (GISAID, https://www.gisaid.org) and are listed in Supporting Table S1. All sequences were aligned using Multiple Alignment using Fast Fourier Transform (MAFFT Version 6.864) software. The genetic analysis was based on mutations causing AA substitutions. Using MEGA Version 11 software, the sequences of the eight segments obtained were compared to the virus sequence (A/Darwin/6/2021) (H3N2) (reference strain for the 2023–2024 influenza vaccine) as presented in Supporting Table S1. The phenotypic properties of eacvdh identified mutation were determined using MEGA software. Phylogenetic trees for the HA, NA and M genes were generated using the maximum likelihood (ML) method with MEGA (Version 11) and visualized with FigTree (Version 1.4.4), using the study sequences, reference sequences and representative sequences from other regions. In addition, a similarity analysis was performed between the genes and encoded proteins of the seasonal influenza H3N2 virus in Cameroon from 2023 to 2024 and strain A/Darwin/6/2021, a vaccine strain of the seasonal influenza H3N2 virus from the northern hemisphere. The robustness of the tree topology was assessed using 1000 bootstrap replications, with values above 70% indicated on the branches of the tree. All sequence data analyzed in this study have been deposited in the GISAID repository, and the accession numbers are detailed in Supporting Table S1.

### 2.5. Classification of Subclades by Amino Acid Substitutions in HA

All sequences were aligned to the selected reference strain. The reference strain used in this study corresponded to the WHO-recommended vaccine strain for the Northern Hemisphere for 2023–2024, which was retrieved from the GISAID database. The subclades of the 33 A/H3N2 strains in this study were determined by key AA substitutions in HA that define A/H3N2 subclades, as proposed by the WHO. Key AA substitutions based on A/Darwin/6/2021, a 2024 Northern Hemisphere vaccine strain, were used to classify the HA 3C.2a group comprising 3C.2a1.2a.2a.1b, 3C.2a1b.2a.2b, 3C.2a1b.1a, and 3C.2a1b.2a.2a.3a.1. The subclassification of vaccine strains for each year was performed based on HA substitutions. Genetic analysis of AA substitutions was performed using MEGA V.7.0.26 software (https://www.megasoftware.net/; accessed on July 17, 2025). In order to better understand the classification of clades and the grouping of viruses, a phylogenetic tree was constructed from 67 strains from other countries around the world. These were then compared to vaccine strains from the Northern Hemisphere belonging to known clades recommended by the WHO.

### 2.6. 3D Structure of HA, NA, and MP Proteins

In order to highlight the AA substitutions observed in the three genes, the codes for the HA, NA, and NA proteins, 4O5N, 1ING, and 8RNG, respectively, were uploaded to the Protein Data Bank (PDB) along with the various trimeric and tetrameric structures corresponding to these proteins. These three structures were then imported into the PyMOL software for visualization and annotation of the observed AA substitutions. The transparency of the contours and the surface was adjusted to 80%.

## 3. Results

### 3.1. Description of the Socio-Demographic Characteristics of the Study Population

Using stratified random sampling, 33 real-time PCR-positive nasopharyngeal specimens from influenza patients with Ct < 30 were selected and included in our study. Of these samples, 30 (90%) were from individuals attending outpatient clinics (ILI), while 3 (09%) were from hospitalized patients (SARI). Patients ranged in age from 1 to 90 years, with 19 out of 33 (57.6%) being female. Myalgias, cough, and vomiting were the major symptoms observed in 90% of the patients included in this study. Influenza A (H3N2) viruses were identified from both outpatients and inpatients. In contrast, the 50–64 and over-65 age groups were only slightly affected by influenza infection during this period. Additionally, the predominance of A/H3N2 cases was greatest in the 5–14 age group (18/33), followed by the 0–1 age group (10/33). The majority of influenza cases were observed during the influenza period from October to December of both study years.

### 3.2. Nucleotide Diversity of Influenza A/H3N2 Viruses

For the first time ever in Cameroon, 33 complete genomes of influenza A (H3N2) viruses have been successfully sequenced. Similarity analyses showed that the nucleotide similarity of eight gene fragments in the sequences of the 33 strains ranged from 98.1% to 100%. Compared to the A/Darwin/6/2021 strain, the nucleotide similarity of the eight gene segments ranged from 98.1% to 100%. AA similarity among the encoded proteins varied between 97.3% and 100%, as shown in Table 1. In summary, this study provides clear information on the genetic characteristics and evolutionary distances of influenza A (H3N2) viruses, which will be used to select new vaccine strains.

### 3.3. Analysis of Genetic Variation and Evolution of H3N2 Influenza Viruses

**Figure 1:**
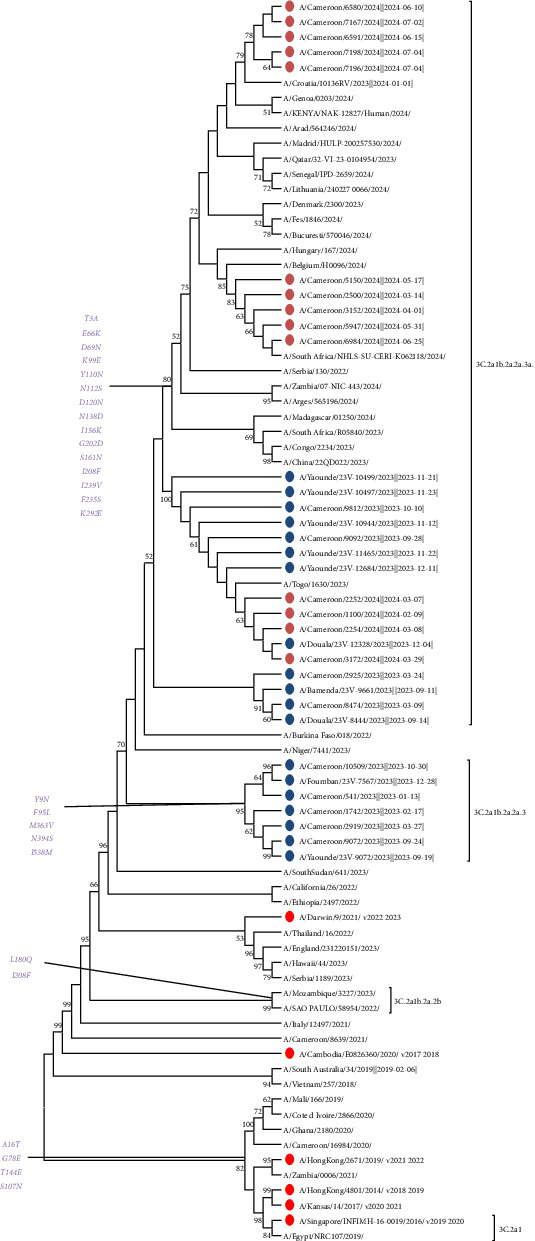
HA gene phylogeny of influenza A/H3N2 viruses detected in Cameroon during the 2023-2024 season. The vaccine reference strains for the northern hemisphere are shown in red. The Cameroonian viruses detected during the 2023 and 2024 seasons are shown in blue and brown, respectively. All viruses were detected in unvaccinated outpatients and hospitalized patients. Phylogenetic trees for the HA genes were generated using the maximum likelihood (ML) method with MEGA (Version 11). The robustness of the tree topology was assessed with 1000 bootstrap replicates, with values above 70% indicated on tree branches.

During our study, the genetic evolution of A/H3N2 influenza viruses in Cameroon was analyzed using the sequences of their eight segments. For the segments (HA, NA, and M), a phylogenetic tree was constructed from the sequences of the eight segments of 33 A/H3N2 strains, including the global vaccine strain recommended by the WHO (2023–2024) and strains from other countries. The results revealed that all sequences from these eight segments were closely related and located in the same clade, 3c.2a. It should be noted that the HA genes from these sequences all belonged to subclade 3c.2a1b.2a.2a.3a.1. The remaining integrated strains evolved in a distinct evolutionary clade, with their HA genes belonging to subclades 3c.2a1b.2a.2a.1b, 3c.2a1b.2a.2b, and finally to subclade 3c.2a1b.1a (Figure 1). Overall, phylogenetic analysis of the eight-segment sequences of influenza A/H3N2 viruses in Cameroon revealed four distinct evolutionary subclades, with the HA genes of the sequences belonging either to subclade 3c.2a1b.2a.2a.1b, or subclade 3c.2a1b.2a.2b, or subclade 3c.2a1b.1a, and finally subclade 3c.2a1b.2a.2a.3a.1.

### 3.4. Subclade Analysis by AA Substitutions and Phylogenetic Tree Analysis of HA, NA, and MP Genes

**Figure 2:**
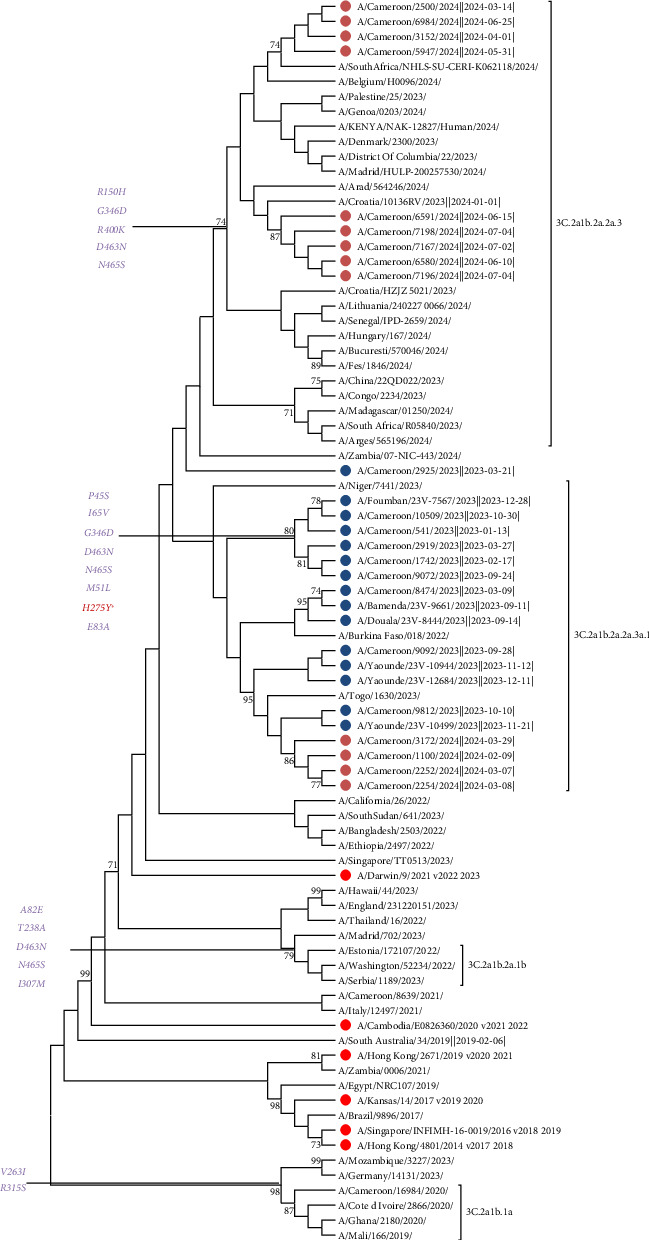
NA gene phylogeny of influenza A/H3N2 viruses detected in Cameroon during the 2023-2024 seasons. The vaccine reference strains for the northern hemisphere are shown in red. The Cameroonian viruses detected during the 2023 and 2024 seasons are shown in blue and brown, respectively. All viruses were detected in unvaccinated outpatients and hospitalized patients. Phylogenetic trees for the NA genes were generated using the maximum likelihood (ML) method with MEGA (Version 11). The robustness of the tree topology was assessed with 1000 bootstrap replicates, with values above 70% indicated on tree branches.

**Figure 3:**
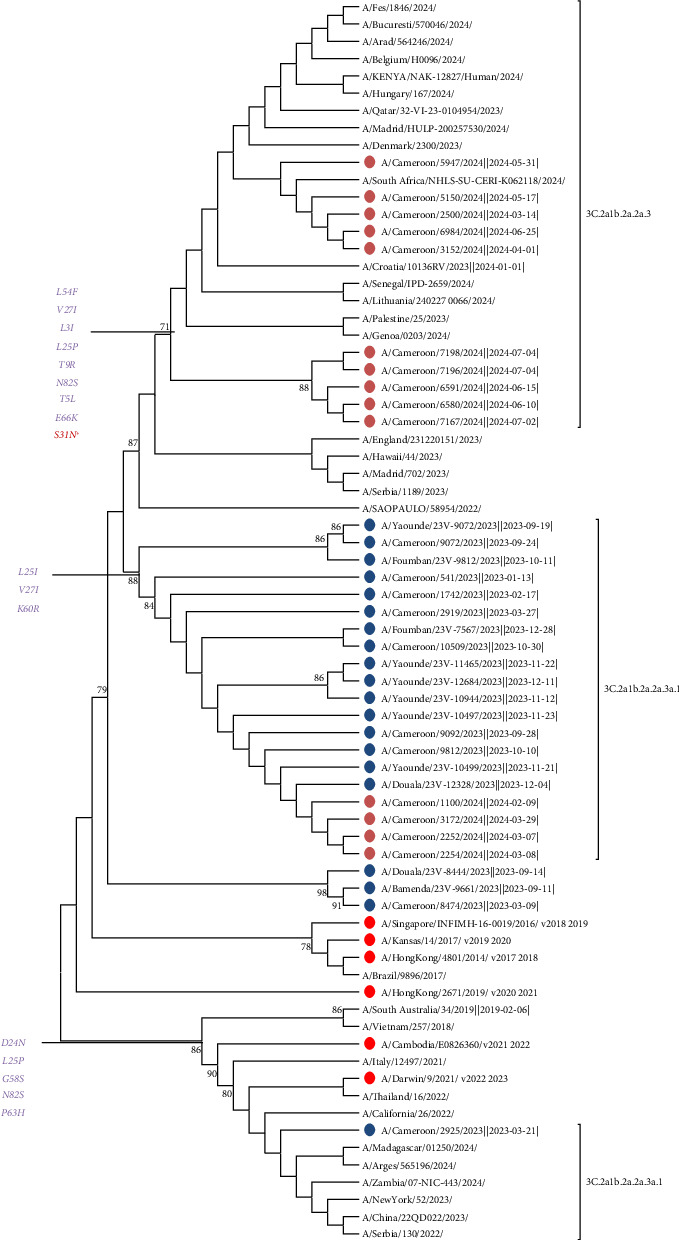
Phylogeny of the MP gene of influenza A/H3N2 viruses detected in Cameroon during the 2023-2024 seasons. The vaccine reference strains for the northern hemisphere are shown in red. The Cameroonian viruses detected during the 2023 and 2024 seasons are shown in blue and brown, respectively. All viruses were detected in unvaccinated outpatients and hospitalized patients. Phylogenetic trees for the MP genes were generated using the maximum likelihood (ML) method with MEGA (Version 11). The robustness of the tree topology was assessed with 1000 bootstrap replicates, with values above 70% indicated on tree branches.

The 33 strains from Cameroon all had AA substitutions at sites I208F, K156I, E66K, N112S, G69N, V239I, K292E, Q189R, G241D, A202D, T3A, S70R, N161S, N138D, N394S, N120D, A16T, G78E, and T144E in HA1 compared to the 2024 vaccine strain A/Darwin/6/2021. The results indicated that the 33 A/H3N2 viruses from Cameroon collected between 2023 and 2024 belonged to clade 3C.2a. Of the 33 strains, analysis of AA substitutions in HA showed that the majority of strains from 2023 to 2024 belonged to subclade 3c.2a1b.2a.2a.3a.1 (*n* = 70%). The remaining strains belonged to subclade 3C.2a1b.2a.2a.3 (*n* = 30%). All of the specific AA mutations in HA1, which define the subclades relative to the northern hemisphere vaccine strain A/Darwin/6/2021, are summarized in Supporting Table S2. Phylogenetic analysis of HA confirmed that all Cameroon sequences grouped in clade 3c.2a1b.2a.2a.3a.1 evolved separately from the reference vaccine strain for the 2023–2024 influenza season (Figure 1) and its subclades according to the AA defined in HA by the WHO. Furthermore, the number of AA mutations observed between circulating strains and the vaccine strain for the influenza season was higher, which also indicates that the circulating strains were different from the recommended vaccine strain. In order to evaluate the genetic relationship between the strains from Cameroon and those circulating worldwide, we performed a BLAST search for the HA gene of 67 A/H3N2 sequences and constructed a phylogenetic tree. The HA gene in Cameroon was closest to strains isolated in South Africa, Togo, Burkina Faso, Niger, China, Belgium, and Italy between 2023 and 2024 (Figure 1). We also noted that the A/H3N2 viruses from 2023 to 2024 in Cameroon, belonging respectively to subclades 3c.2a1b.2a.2a.3a.1 and 3C.2a1b.2a.2a.3, were closely related to subclade 3C.2a1b.2a. 2b. Similarly, AA substitutions at sites I469T, I65V, P45S, I392T, L140I, L338V, H150R, K400R, and H275Y in the NA protein were observed compared to the vaccine strain 2024 A/Darwin/6/2021. We noted the presence of the H275Y substitution in 30% of Cameroonian strains associated with major resistance to NA inhibitors, specifically oseltamivir. Phylogenetic analysis of NA confirmed that all Cameroonian sequences grouped in clade 3c.2a1b.2a.2a.3a.1 evolved separately from the reference vaccine strain for the 2023–2024 influenza season (Figure 2). In addition, AA substitutions at sites N85S, L59I, D24N, V27I, Y52C, F54L, L25P, S82N, E66K, and S31N in the MP protein were observed compared to the vaccine strain 2024 A/Darwin/6/2021. We noted the presence of the major substitution S31N in some Cameroonian strains, which is known to confer a high level of resistance to amantadine. All of the specific AA mutations in NA are summarized in Supporting Table S3. Phylogenetic analysis of MP confirmed that all but a few of the Cameroonian sequences grouped in clade 3c.2a1b.2a.2a.3a.1 evolved separately from the reference vaccine strain (Figure 3). All of the specific AA mutations in MP are summarized in Supporting Table S4.

### 3.5. Analysis of Comparison of AA Substitutions Between Circulating Strains and A/Darwin/6/2021 Vaccine Strain in Eight Genes

Compared to the 2023–2024 vaccine strain A/Darwin/6/2021, the mutations I208F, K156I, E66K, N112S, G69N, V239I, K292E, Q189R, G241D, A202D, T3A, S70R, N161S, N138D, N394S, A16T, G78E, and N120D were detected in most samples of HA1 genes (Supporting Table S2). Among these mutations, the important A202D and N161S mutations in HA1 in 2023 and 2024 led to virus virulence and consequently conferred rapid evolution of the A/H3N2 virus and the generation of the new clades 3C.2a1b.2a.2a.3 and 3c.2a1b.2a.2a.3a.1, respectively. Similarly, mutational analyses were extended to the seven longest viral genes (PB2, PB1, PA, NS, and NP; Supporting Tables S5–S9). In the NP gene, a total of 9 AA substitutions (E220D, L418I, L136M, I186V, A129S, N432S, S482N, K236R, and S359L) were observed. In the PA gene, a total of 18 AA substitutions (S402A, G684R, K142N, E101G, M311I, R605K, K142N, R213K, G99E, A660S, I407S, C321Y, A20T, K269R, K497R, R158K, Y277H, and L400I) were observed. Similarly, new AA substitutions not previously described have been observed in the PB1 gene (V200I, S375N, R386K, and V527I) and PB2 (R340K, D87N, V461I, N107D, T147I, M410V, and L384F). Numerous mutations have also been identified in the NS genes, notably the substitutions E152D, N207H, I18V, N127S, N26K, A60V, R227G, I33L, I124M, A82V, V171I, L14S, and K88R. In general, the number of AA mutations observed between circulating strains and the vaccine strain for the following year was higher, indicating that circulating strains would evolve away from the vaccine strains for the year 2023–2024.

### 3.6. 3D Structure of HA, NA, and MP Proteins

**Figure 4:**
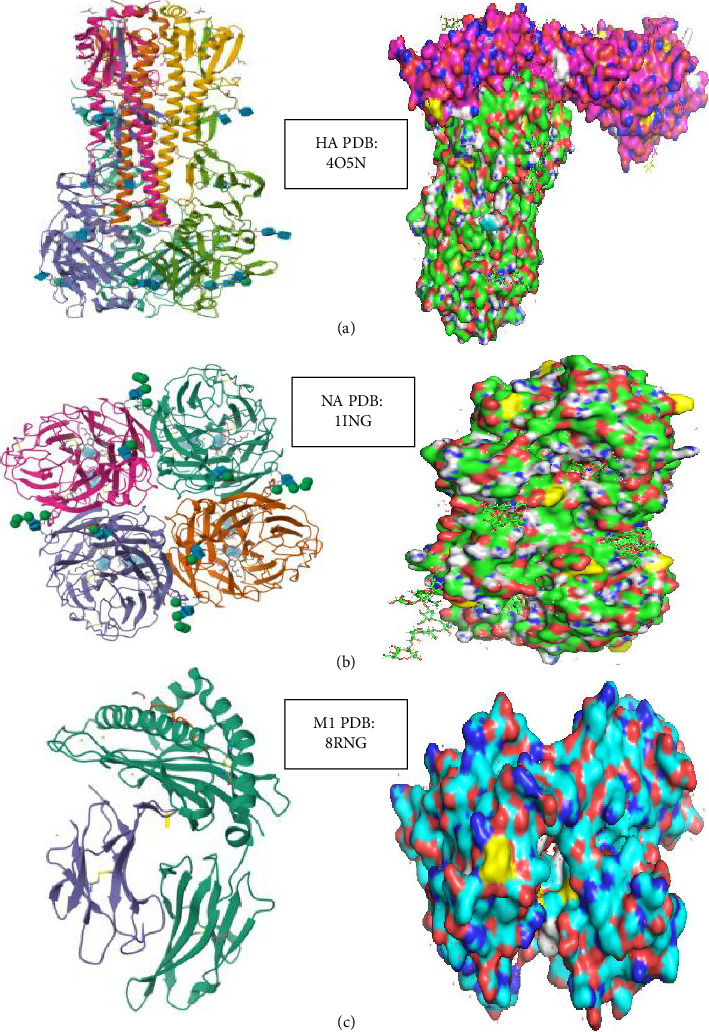
Epitopes of the HA, NA, and M1 H3 head domains and amino acid sequences of mutant epitope substitutions. (a) Crystal structure of the HA H3 trimer (PDB: 4O5N) with the classically defined antigenic sites colored as follows: Sa in red, Sb in green, Ca1 in blue, Ca2 in magenta, and Cb in orange. (b) Crystal structure of the NA H3 tetramer (PDB: 1ING). (c) Crystal structure of the M1 H3 trimer (PDB: 8RNG). Modeling was performed with PyMOL (the PyMOL Molecular Graphics System, Version 2.0.1, Schrödinger, LLC). A sialic acid molecule is present in the binding pocket of the pink HA monomer receptor. The amino acid sequences of the heterologous epitopes for the panel of mutant viruses are listed under the respective H3 sites. Amino acids in white and yellow represent substituted residues. Amino acids in grey and blue are unchanged.

The three-dimensional structures of the HA, NA, and MP proteins from sequences in clade 3C.2a1b.2a.2a.3a.1 were modeled. The HA protein exhibited several mutations, including I208F, K156I, E66K, N112S, G69N, V239I, K292E, Q189R, A202D, T3A, S70R, N161S, N138D, N394S, N120D, and G241D. In the NA protein, mutations such as I469T, P45S, I65V, H275Y, I392T, L140I, L338V, H150R, and K400R were identified. Additionally, the M protein displayed variations at sites N85S, L59I, D24N, V27I, Y52C, S31N, F54L, L25P, S82N, and E66K. The variation sites were distinctly marked in the three-dimensional structures with different colors, as illustrated in Figure 4.

## 4. Discussion

Several studies have utilized whole influenza virus genome sequences and modern software to understand the evolutionary dynamics of the influenza A/H3N2 subtype [11–13]. The replication of the RNA genome in influenza viruses is associated with a relatively high mutation rate (2.3 × 10^−5^), primarily because the viral RNA-dependent RNA polymerase lacks 3′–5′-exonuclease activity, leading to an absence of proofreading functions [14, 15]. This study represents the second report of genome characterization of influenza H3N2 in Cameroon, following an earlier study by Monamele et al. [16] that focused on partial segments of the HA, NA, and M genes from 2014 to 2016. The WHO influenza established a global surveillance network and recommends annual influenza vaccine strains for the northern and southern hemispheres. Cameroon is one of the most crucial member countries for influenza surveillance and has established a surveillance network covering all cities in the country.

In the HA genes of the isolated strains, significant substitutions such as E66K, I208F, N112S, N394S, G241D, G69N, K156I, V239I, K292E, Q189R, N175S, R108K, G291D, and A202D were observed. Among these mutations, the important A202D and N161S mutations in HA1 in 2023 and 2024 led to virus virulence and consequently conferred rapid evolution of the A/H3N2 virus and the generation of the new clades 3C.2a1b.2a.2a.3 and 3c.2a1b.2a.2a.3a.1. These findings contrast with those observed in southern China in 2012, where the D69N, Y110H, I246V, and E296A/T substitutions involved in the rapid evolution of the A/H3N2 virus were identified [17]. The observed mutational diversity from year to year may be attributed to AA changes at sites associated with human leukocyte antigen (HLA), which alter the HA antiglobulin antibody recognition sites.

Similarly, the mutations involved in the virulence of Cameroonian strains circulating between 2023 and 2024 differ from those reported in 2017 by Monamele et al., who identified the mutations N145S, Y186G, P198S, and F219S in the HA polypeptide. This variability highlights the significant antigenic diversity that exists within the binding sites of the HA gene. This antigenic variability observed within the HA gene can also be confirmed by the results obtained by Ramuth et al. in 2025, which showed significant antigenic diversity following the cocirculation of subclades 3C.2a4 and 3C.2a1 in 2017, while the predominant subclade in 2018 was subclade 3C.2a1b.1. Phylogenetic analysis of the HA gene of A(H3N2) viruses showed that the vast majority of viruses circulating in Cameroon during the 2023–2024 influenza season belonged to clade 2 (full classification 3C.2a1b.2a.2) and had acquired several AA substitutions. The different HA subclades were found in different regions of the world, and viruses with HA genes from several subclades cocirculated in several geographical regions in varying proportions. Among the viruses with HA genes from clade 2 that cocirculated during this period, three subclades predominated: 2a.1b (generally encoding D53G, D104G, I140K, K276R, and R299K), 2a.3a.1 (generally encoding E50K, G53N, N96S (CHO+), I140K, I192F, I223V, and N378S), and 2b (generally encoding E50K, F79V, and I140K). Among these, subclade 2a.3a.1, which includes more than 70% of Cameroonian strains (e.g., A/Massachusetts/18/2022), was predominant worldwide. These viruses were detected mainly in Africa, Asia, North America, and Oceania. No Cameroonian strains were grouped in subclade 2a.1b, which was detected mainly in North America and Europe, as shown in Figure 1. Clade 2b viruses circulated globally. In general, postinfection ferret antisera produced against SH 2023 vaccine viruses (A/Darwin/6/2021 viruses propagated in cell culture and A/Darwin/9/2021 2a viruses propagated in eggs) recognized viruses expressing HA 2a genes (including subclades) well. However, some viruses expressing HA 2a.3a.1 genes, such as the majority of Cameroonian strains (70%), reacted less well with these antisera during the 2023–2024 influenza season. This evolution of clade 2a.3a.1 viruses away from the A/Darwin/6/2021 vaccine reference virus was also observed in 2017 by Monamele et al., who showed that as the 2012 flu season approached, the HA gene sequences indicated that all Cameroonian strains had evolved away from the 3C.1-A/Darwin/6/2021 clade. This observation was confirmed in 2019 in northern Cameroon by Njifon et al. [18] and also in 2022 in the city of Myanmar, Japan, by Phyu et al., who observed that the Myanmar strains differed from the Southern Hemisphere vaccine strains each year, indicating a mismatch between the vaccine strains and the circulating strains [19]. These results update the findings of Monamele et al., who showed in 2017 that Cameroonian strains formed two distinct groups. These illustrations indicate that the molecular characterization of the influenza virus varies from year to year, region to region, and country to country, as several authors have pointed out [20–22]. Furthermore, the NA and M strains of influenza from 2023 to 2024 did not cluster with any strain but closely resembled the 2019–2020, 2021–2022, and 2022–2023 strains (3C.3a-A/Kansas/14/2014, 3C.2a-A/Cambodia/E0826360/2020, and 3C.2a-A/Darwin/9/2021, respectively). This observation aligns with Monamele et al., who indicated that the Cameroonian NA and M gene sequences were similar to those of the reference strains, evolving away from the 3C.1-A/Texas/50/2012 clade and acquiring several AA substitutions.

In 2011, a Canadian study looking at the antigenic and molecular characterization of H3N2 viruses over three seasons revealed significant HA mutations, as well as the nature and location of the main mutations, which played a crucial role in antigenic drift. This study again confirms the significant mutational variation observed in the HA binding sites of Cameroonian strains. Furthermore, the results of our study showed that 30% of Cameroonian strains, that is, 10/33, had a point mutation (cytosine to thymidine) at position 823, which causes the substitution of histidine by tyrosine at position 275 in the AA sequence of NA (H275Y). This substitution has been reported by Pinella et al. as conferring more than 1.5% resistance to oseltamivir in patients with ARI. We also noted the absence of the S31N substitution present in the M2 protein of many previous Cameroonian strains, which conferred resistance to amantadine. This resistance was confirmed in studies described by [23, 24]. Our data instead confirmed the presence of the V27I mutation, which causes strong phenotypic resistance to amantadine at a level similar to that of the S31N mutation. This result is similar to that of Balannik et al., who reported that the S31D mutation reduced the ability of amantadine to block the M2 channel to a comparable level as the S31N mutation [25]. Monitoring amantadine resistance among A (H3N2) viruses from 1991 to 2024 revealed that the global incidence of resistance was well above 50%, while a recent study showed that the incidence of resistance had reached 96% and 72% in China and South Korea, respectively. This consistent increase in the frequency of resistance to amantadine in Asian countries sharing a people-flow border with Cameroon emphasizes the importance of incorporating full genome sequences alongside antigenic data to predict which influenza strains are likely to prevail in the upcoming influenza seasons [26]. Given that the number of antiviral drugs available to treat and prevent influenza virus infections is very limited, it is very important to understand the mechanisms that cause resistance to INAs and to establish a program to monitor the evolution of antiviral-resistant strains.

## 5. Conclusions

The presence of numerous substitution mutations in most of the Cameroonian strains isolated in 2024, which were absent in the 2023 strains, clearly indicates that the A/H3N2 strains circulating in Cameroon are constantly evolving. This finding highlights the need for systematic genomic surveillance of seasonal influenza viruses to assess the burden of influenza infections and to adopt effective national influenza control and prevention strategies.

## Figures and Tables

**Table 1:** Similarity analysis of the complete genome of the influenza A (H3N2) virus in Cameroon.

Genes	Proteins	Sequence similarity between 33 strains (%)		Similarity compared to A (H3N2) (%)	
		Nucleotides	Amino acid	Nucleotides	Amino acid
HA	HA	99.4–100	99.4–100	99.4–99.8	99.4–99.9

NA	NA	99.7–100	99.7–100	99.7–99.0	98.7–98.9

M	M1	98.1–100	98.1–100	98.1–100	97.1–99.0
	M2	97.1–100	97.3–100	97.1–98.5	95.1–98.0

NP	NP	98.7–100	98.7–100	98.7–100	98.7–98.9

NS	NS1	99.3–100	99.3–100	99.3–100	99.4–99.6
	NS2	99.8–100	99.8–100	98.8–99.1	99.8–100

PA	PA	98.9–100	98.9–99.6	98.9–99.5	98.9–99.7

PB1	PB1	99.5–100	99.5–100	99.5–99.8	97.5–98.5

PB2	PB2	99.1–100	99.1–100	97.1–98.2	99.1–100

## Data Availability

The authors confirm that the data supporting the findings of this study are available within the article.

## Supporting Information

Supporting Information 1 Supporting Table S1: GISAID isolate ID.

Supporting Information 2 Supporting Table S2: List of mutation differences in the HA gene between Cameroon 2023-2024 viruses and the A/Darwin/6/2021 vaccine strain.

Supporting Information 3 Supporting Table S3: List of mutation differences in the NA gene between Cameroon 2023-2024 viruses and the A/Darwin/6/2021 vaccine strain.

Supporting Information 4 Supporting Table S4: List of mutation differences in the NP gene between Cameroon 2023-2024 viruses and the A/Darwin/6/2021 vaccine strain.

Supporting Information 5 Supporting Table S5: List of mutation differences in the NS gene between Cameroon 2023-2024 viruses and the A/Darwin/6/2021 vaccine strain.

Supporting Information 6 Supporting Table S6: List of mutation differences in the PA gene between Cameroon 2023-2024 viruses and the A/Darwin/6/2021 vaccine strain.

Supporting Information 7 Supporting Table S7: List of mutation differences in the PB1 gene between Cameroon 2023-2024 viruses and the A/Darwin/6/2021 vaccine strain.

Supporting Information 8 Supporting Table S8: List of mutation differences in the PB2 gene between Cameroon 2023-2024 viruses and the A/Darwin/6/2021 vaccine strain.

Supporting Information 9 Supporting Table S9: List of mutation differences in the MP gene between Cameroon 2023-2024 viruses and the A/Darwin/9/2021 vaccine strain.
