# Supplementary material for: Evolving Dynamics of Whole-Genome Influenza A/H3N2 Viruses Isolated in Cameroon
Source: Adv Virol. 2025 Sep 19;2025:3668615. doi: 10.1155/av/3668615 (PMC12473741; doi:10.1155/av/3668615)
Supplement: Supporting Information 1 — Supporting Table S1: GISAID isolate ID. [file 3668615.f1.docx]

| **NAME** | **GISAID ID** |
| --- | --- |
| A/Cameroon/5947/2024 | EPI_ISL_19387253 |
| A/Cameroon/5150/2024 | EPI_ISL_19387237 |
| A/Cameroon/2500/2024 | EPI_ISL_19387043 |
| A/Cameroon/6984/2024 | EPI_ISL_19387249 |
| A/Cameroon/3152/2024 | EPI_ISL_19387212 |
| A/Cameroon/7196/2024 | EPI_ISL_19387198 |
| A/Cameroon/7198/2024 | EPI_ISL_19387215 |
| A/Cameroon/6591/2024 | EPI_ISL_19387230 |
| A/Cameroon/6580/2024 | EPI_ISL_19387215 |
| A/Cameroon/7167/2024 | EPI_ISL_19387223 |
| A/Bamenda/23V-9661/2023 | EPI_ISL_19176224 |
| A/Douala/23V-8444/2023 | EPI_ISL_19176222 |
| A/Cameroon/8474/2024 | EPI_ISL_19085889 |
| A/Cameroon/10509/2023 | EPI_ISL_118991095 |
| A/Bamenda/23V-9812/2023 | EPI_ISL_19176225 |
| A/Yaounde/23V-9072/2023 | EPI_ISL_19176223 |
| A/Foumban/23V-7567/2023 | EPI_ISL_19176221 |
| A/Cameroon/2919/2023 | EPI_ISL_18167523 |
| A/Cameroon/1742/2023 | EPI_ISL_18167526 |
| A/Cameroon/541/2023 | EPI_ISL_18167527 |
| A/Yaounde/23V-11465/2023 | EPI_ISL_19176230 |
| A/Yaounde/23V-12684/2023 | EPI_ISL_19176223 |
| A/Yaounde/23V-10944/2023 | EPI_ISL_19176229 |
| A/Yaounde/23V-10497/2023 | EPI_ISL_19176226 |
| A/Cameroon/9092/2023 | EPI_ISL_19085888 |
| A/Yaounde/23V-10499/2023 | EPI_ISL_19176227 |
| A/Douala/23V-10940/2023 | EPI_ISL_19176228 |
| A/Douala/23V-12328/2023 | EPI_ISL_19176233 |
| A/Cameroon/1100/2024 | EPI_ISL_19387265 |
| A/Cameroon/3172/2024 | EPI_ISL_19387265 |
| A/Cameroon/2254/2024 | EPI_ISL_19387261 |
| A/Cameroon/2252/2024 | EPI_ISL_19387227 |
| A/Cameroon/2925/2023  A/Darwin/9/2021 | EPI_ISL_18167525  EPI_ISL_12109641 |

**Supplemental Table 1**. GISAID Isolate ID
