# Supplementary material for: Evolving Dynamics of Whole-Genome Influenza A/H3N2 Viruses Isolated in Cameroon
Source: Adv Virol. 2025 Sep 19;2025:3668615. doi: 10.1155/av/3668615 (PMC12473741; doi:10.1155/av/3668615)
Supplement: Supporting Information 2 — Supporting Table S2: List of mutation differences in the HA gene between Cameroon 2023-2024 viruses and the A/Darwin/6/2021 vaccine strain. [file 3668615.f2.docx]

**Supplementary Table S2**: List of mutation differences in the HA gene between Cameroon 2023–2024 viruses and the A/Darwin/6/2021 vaccine strain

| **Virus Strain** | **HA1** |  |  |  |  |  |  |  |  |  |  |  |
| --- | --- | --- | --- | --- | --- | --- | --- | --- | --- | --- | --- | --- |
|  | 3 | 17 | 18 | 19 | 20 | 22 | 37 | 39 | 66 | 69 | 70 | 88 |
| **A/Darwin/6/2021(H3N2** | T | - | K | N | T | K | T | R | K | N | R | G |
| A/Cameroon/1100/2024 | . | - | . | . | . | . | . | . | . | Q | . | . |
| A/Cameroon/2252/2024 | . | - | . | . | . | . | . | . | . | Q | . | . |
| A/Douala/23V-12328/2023 | . | - | . | . | . | . | . | . | . | Q | .S | . |
| A/Yaounde/23V-11465/2023 | . | - | . | . | . | . | . | . | . | Q | . | . |
| A/Yaounde/23V-10497/2023 | . | - | . | . | . | . | . | . | . | Q | . | . |
| A/Yaounde/23V-12684/2023 | . | - | . | . | . | . | . | . | . | G | . | . |
| A/Cameroon/9092/2023 | . | - | . | . | . | . | . | . | . | Q | . | . |
| A/Cameroon/9812/2023 | . | - | . | . | . | . | . | W | . | Q | . | . |
| A/Yaounde/23V-10499/2023 | . | - | . | . | . | . | . | . | . | Q | . | . |
| A/Cameroon/3172/2024 | . | - | . | . | . | . | . | . | . | Q | . | . |
| A/Bamenda/23V-9661/2023 | . | - | . | . | . | . | . | . | . | Q | .S | . |
| A/Cameroon/8474/2023 | . | - | . | . | . | . | . | . | . | Q | . | . |
| A/Cameroon/2925/2023 | A | - | . | . | . | . | . | . | . | Q | . | . |
| A/Douala/23V-8444/2023 | . | - | . | K | P | . | . | . | . | . | . | . |
| A/Cameroon/10509/2023 | . | - | R | . | . | . | . | . | . | G | . | . |
| A/Cameroon/541/2023 | . | - | . | . | . | . | . | . | . | Q | . | . |
| A/Cameroon/1742/2023 | . | - | . | . | . | . | . | . | . | Q | . | . |
| A/Cameroon/2919/2023 | . | - | . | . | . | . | . | . | . | Q | . | . |
| A/Yaounde/23V-9072/2023 | . | - | . | . | . | R | . | . | . | Q | . | . |
| A/Foumban/23V-7567/2023 | . | P | . | . | . | . | . | . | . | Q | . | . |
| A/Cameroon/5150/2024 | A | - | . | . | . | . | . | . | . | Q | A | R |
| A/Cameroon/2500/2024 | A | - | . | . | . | . | . | . | . | Q | A | R |
| A/Cameroon/5947/2024 | A | - | . | . | . | . | . | . | . | Q | A | R |
| A/Cameroon/6984/2024 | A | - | . | . | . | . | . | . | . | Q | A | R |
| A/Cameroon/3152/2024 | A | - | . | . | . | . | . | . | . | Q | A | R |
| A/Cameroon/7196/2024 | A | - | . | . | . | . | A | . | E | Q | . | . |
| A/Cameroon/7198/2024 | A | - | . | . | . | . | A | . | G | Q | . | . |
| A/Cameroon/6580/2024 | A | - | . | . | . | . | A | . | G | Q | . | . |
| A/Cameroon/6591/2024 | A | - | . | . | . | . | A | . | G | Q | . | . |
| A/Cameroon/7167/2024 | A | - | . | . | . | . | A | . | G | Q | . | . |
| 90 | 91 | 112 | 106 | 120 | 121 | 126 | 128 | 130 | 133 | 138 | 145 | 149 |
| P | S | S | K | D | L | V | S | L | H | D | W | K |
| S | P | . | T | . | . | . | G | . | N | . | R | . |
| S | P | . | T | . | . | . | G | . | N | . | R | . |
| S | P | . | T | .N | . | . | G | . | N | .N | R | . |
| S | P | . | T | . | . | . | G | . | N | . | R | . |
| S | P | .N | T | . | . | . | G | . | N | . | R | . |
| S | P | . | T | . | . | . | G | . | N | . | R | . |
| S | P | . | T | .N | . | . | G | . | N | . | R | . |
| S | P | . | T | . | . | . | G | . | N | . | R | . |
| S | P | . | T | . | . | . | G | . | N | .N | R | . |
| S | P | . | T | . | . | . | G | . | N | . | R | . |
| . | . | . | T | . | . | . | . | . | N | . | R | . |
| . | . | . | T | . | . | . | . | . | N | . | R | . |
| . | . | . | T | . | . | . | . | . | N | . | R | . |
| . | . | . | T | . | . | . | . | . | N | . | S | . |
| . | . | . | T | .N | . | I | . | . | N | . | R | . |
| . | . | . | T | . | . | . | . | . | N | . | R | . |
| . | . | . | T | . | . | . | . | . | N | . | R | . |
| . | . | . | T | . | . | . | . | . | N | N | R | . |
| . | . | . | T | . | . | . | . | . | N | . | R | . |
| . | . | . | T | . | . | I | . | . | N | . | R | . |
| . | . | E | T | . | . | . | . | F | N | R | R | . |
| . | . | E | T | . | . | . | . | F | N | R | R | . |
| . | . | E | T | . | . | . | . | F | N | R | R | . |
| . | . | E | T | . | S | . | . | F | N | R | R | . |
| . | . | E | T | . | . | . | . | F | N | R | R | . |
| . | . | E | T | E | . | . | . | . | N | R | R | E |
| . | . | E | T | E | . | . | . | . | N | R | R | E |
| . | . | E | T | E | . | . | . | . | N | R | R | E |
| . | . | E | T | E | . | . | . | . | N | R | R | E |
| . | . | E | T | E | . | . | . | . | N | R | R | E |
| 156 | 161 | 180 | 183 | 189 | 200 | 202 | 204 | 206 | 208 | 209 | 214 | 218 |
| I | S | T | D | R | P | D | G | E | F | L | I | N |
| K | . | A | . | . | . | . | . | . | I | . | . | . |
| Q | . | A | . | . | . | . | . | . | I | . | . | . |
| Q | .N | A | . | . | . | . | . | . | I | . | . | . |
| Q | . | . | . | . | . | . | . | . | I | . | . | . |
| Q | . | . | . | .Q | . | . | . | . | I | . | . | . |
| K | . | . | . | . | . | . | . | . | I | . | . | . |
| Q | N | . | . | . | . | A | . | . | I | . | . | . |
| Q | . | . | . | . | . | . | . | . | I | . | . | . |
| Q | . | . | . | . | . | . | . | G | I | . | V | . |
| Q | . | A | . | . | . | . | . | . | I | . | . | . |
| Q | P | . | . | . | . | . | . | . | I | . | . | . |
| Q | . | . | . | . | . | . | . | . | I | . | . | . |
| Q | . | . | N | . | . | . | R | . | I | . | . | . |
| Q | . | . | . | . | . | . | . | . | I | . | . | . |
| . | . | . | . | H | . | . | . | . | I | . | . | . |
| . | . | . | . | H | . | . | . | . | I | . | . | . |
| . | . | . | . | H | . | . | . | . | I | . | . | . |
| . | . | . | . | H | . | . | . | . | I | . | . | . |
| . | . | . | . | H | . | . | . | . | I | F | . | D |
| . | . | . | . | H | . | . | . | . | I | . | . | . |
| Q | . | . | . | . | S | S | . | . | I | . | . | . |
| Q | . | . | . | . | S | S | . | . | I | . | . | . |
| Q | . | . | . | . | S | S | . | K | I | . | . | . |
| Q | . | . | . | . | S | S | . | . | I | . | . | . |
| Q | . | . | . | . | S | S | . | . | I | . | . | . |
| Q | . | . | . | . | S | S | . | . | I | . | . | . |
| Q | . | . | . | . | S | S | . | . | I | . | . | . |
| Q | . | . | . | . | S | S | . | . | I | . | . | . |
| Q | . | . | . | . | . | S | . | . | I | . | . | . |
| Q | . | . | . | . | S | S | . | . | I | . | . | . |
| 239 | 239 | 241 | 258 | 267 | 268 | 271 | 288 | 292 | 293 | 296 | 325 | 338 |
| N | I | D | H | S | N | * | C | E | M | * | C | K |
| S | . | . | . | . | D | . | R | K | . | . | R | . |
| S | . | . | . | . | D | . | R | R | . | . | R | . |
| S | . | . | . | . | D | . | R | R | . | . | R | . |
| S | .V | . | . | . | D | . | R | R | . | . | R | . |
| S | . | .G | . | . | D | . | R | R | . | . | R | . |
| S | . | . | . | . | D | . | R | R | . | W | R | . |
| S | .V | . | R | . | D | . | R | R | . | . | R | . |
| S | . | . | . | . | D | . | R | R | . | . | R | . |
| S | . | . | . | . | D | . | R | R | . | . | R | . |
| S | . | . | . | F | D | . | R | R | . | . | R | . |
| S | . | .G | . | . | . | Q | R | . | . | . | R | . |
| S | . | . | . | . | . | Q | R | . | V | . | R | . |
| S | . | L | . | . | . | . | R | . | . | . | R | . |
| S | . | . | . | . | . | Q | R | . | . | . | R | . |
| . | . | . | . | . | . | . | R | . | . | . | R | E |
| . | . | . | . | . | . | . | R | . | . | . | R | . |
| . | . | . | . | . | . | . | R | . | . | . | R | . |
| . | E | . | . | . | . | . | R | . | . | . | R | . |
| . | . | . | . | . | . | . | R | . | . | . | R | . |
| . | . | . | . | . | . | . | R | . | . | . | R | . |
| S | . | . | . | . | . | . | R | R | . | . | R | . |
| S | . | . | . | . | . | . | R | R | . | . | R | . |
| S | . | . | . | . | . | . | R | R | . | . | R | . |
| S | . | . | . | . | . | . | R | R | . | . | R | . |
| S | . | . | . | . | . | . | R | R | . | . | R | . |
| S | . | . | . | . | . | . | R | R | . | . | R | . |
| S | . | . | . | . | . | . | R | R | . | . | R | . |
| S | . | . | . | . | . | . | R | R | . | . | R | . |
| S | . | . | . | . | . | . | R | R | . | . | R | . |
| S | . | . | . | . | . | . | R | R | . | . | R | . |
| 340 | 341 | 350 | 353 | 368 | 376 | 394 | 397 | 398 | 399 | 402 | 419 | 452 |
| T | R | R | G | R | G | S | A | E | S | R | R | Y |
| A | * | . | . | . | R | . | T | . | . | . | . | . |
| A | * | . | . | . | R | . | T | . | . | . | . | . |
| A | * | . | . | . | R | .N | T | . | . | . | . | . |
| A | W | . | . | . | R | . | T | . | . | . | . | . |
| A | * | . | . | . | R | . | . | . | . | . | . | . |
| A | * | . | . | . | R | . | T | . | . | . | . | . |
| A | * | . | . | . | R | .N | T | . | . | . | . | . |
| A | * | . | . | . | R | . | . | . | . | . | . | . |
| A | * | . | R | . | R | . | . | . | . | . | . | . |
| A | * | . | . | . | R | . | T | . | . | . | . | . |
| . | * | . | . | . | . | . | . | . | . | . | . | . |
| . | * | . | . | . | . | . | . | . | . | . | . | . |
| . | * | . | . | . | . | . | . | . | . | . | . | . |
| . | * | . | R | Q | . | . | . | . | . | W | . | . |
| . | * | . | . | . | . | . | . | . | . | . | G | . |
| . | * | . | . | . | . | . | . | . | . | . | G | . |
| . | * | . | . | . | . | . | . | K | . | . | G | . |
| . | * | . | . | . | . | . | . | K | P | . | G | . |
| . | * | . | . | . | . | . | . | K | . | . | G | H |
| . | * | . | . | . | . | . | . | . | . | . | G | . |
| . | * | C | . | . | . | . | . | . | . | . | . | . |
| . | * | C | . | . | . | . | . | . | . | . | . | . |
| . | * | C | . | . | . | . | . | . | . | . | . | . |
| . | * | C | . | . | . | . | . | . | . | . | . | . |
| . | * | C | . | . | . | . | . | . | . | . | . | . |
| . | * | C | . | . | . | K | . | . | . | . | . | . |
| . | * | C | . | . | . | K | . | . | . | . | . | . |
| . | * | C | . | . | . | . | . | . | . | . | . | . |
| . | * | C | . | . | . | . | . | . | . | . | . | . |
| . | * | C | . | . | . | . | . | . | . | . | . | . |
| 455 | 457 | 463 | 469 | 477 | 483 | 509 | 513 | 516 | 521 | 532 | 550 | 558 |
| P | * | T | E | G | F | G | K | V | S | P | V | G |
| . | . | . | . | . | . | R | . | . | . | . | . | R |
| . | . | . | . | . | . | R | . | . | . | . | . | R |
| . | R | . | . | . | . | R | . | . | . | . | . | R |
| . | . | . | . | . | . | R | . | . | . | . | . | R |
| . | . | . | . | . | . | R | . | . | . | . | . | R |
| . | . | . | . | . | . | R | . | . | . | . | . | R |
| . | . | . | . | . | . | R | . | . | . | . | . | R |
| . | . | . | . | . | . | R | . | . | . | . | . | R |
| . | . | . | . | . | . | R | . | . | . | . | . | R |
| . | . | . | . | . | . | R | N | . | . | . | . | R |
| . | . | . | . | R | . | . | . | . | G | . | . | R |
| . | . | . | . | R | . | . | . | . | G | . | . | . |
| . | . | . | . | . | L | . | . | . | . | . | I | . |
| . | . | . | . | R | . | . | . | . | G | . | . | . |
| S | . | . | K | . | . | . | . | . | . | . | . | . |
| . | . | . | K | . | . | . | . | . | . | . | . | . |
| . | . | . | K | . | . | . | . | . | . | . | . | . |
| . | . | . | K | . | . | . | . | . | . | . | . | . |
| . | . | . | K | . | . | . | . | I | . | . | . | . |
| S | . | . | K | . | . | . | . | . | . | . | . | . |
| . | . | A | . | . | . | . | . | . | . | S | . | . |
| . | . | A | . | . | . | . | . | . | . | S | . | . |
| . | . | A | . | . | . | . | . | . | . | S | . | . |
| . | . | A | . | . | . | . | . | . | . | S | . | . |
| . | . | A | . | . | . | . | . | . | . | S | . | . |
| . | . | A | . | . | . | . | . | . | . | . | . | . |
| . | . | A | . | . | . | . | . | . | . | . | . | . |
| . | . | A | . | . | . | . | . | . | . | . | . | . |
| . | . | A | . | . | . | . | . | . | . | . | . | . |
| . | . | A | . | . | . | . | . | . | . | . | . | . |
