# Supplementary material for: Evolving Dynamics of Whole-Genome Influenza A/H3N2 Viruses Isolated in Cameroon
Source: Adv Virol. 2025 Sep 19;2025:3668615. doi: 10.1155/av/3668615 (PMC12473741; doi:10.1155/av/3668615)
Supplement: Supporting Information 3 — Supporting Table S3: List of mutation differences in the NA gene between Cameroon 2023-2024 viruses and the A/Darwin/6/2021 vaccine strain. [file 3668615.f3.docx]

**Supplementary Table S3**: List of mutation differences in the NA gene between Cameroon 2023–2024 viruses and the A/Darwin/6/2021 vaccine strain

| Virus St | 44 | 45 | 46 | 51 | 56 | 65 | 83 | 94 | 113 | 126 | 140 | 150 | 172 | 215 | 275 | 338 | 356 | 368 | 392 | 400 | 437 | 464 | 469 |
| --- | --- | --- | --- | --- | --- | --- | --- | --- | --- | --- | --- | --- | --- | --- | --- | --- | --- | --- | --- | --- | --- | --- | --- |
| A/Darwin/6/2021(H3N2) NA | S | P | P | M | T | I | E | I | D | L | L | R | K | V | H | L | D | E | I | R | L | L | I |
| A/Cameroon/2925/2023 | . | . | . | . | . | . | . | . | . | . | . | H | . | . |  | . | . | . | . | . | . | . | T |
| A/Cameroon/10509/2023 | . | S | . | . | . | V | . | . | . | . | . | . | . | . | Y | . | . | . | . | . | . | . | T |
| A/Cameroon/541/2023 | . | S | . | . | . | V | . | . | . | . | . | . | . | . |  | . | . | . | . | . | . | . | T |
| A/Foumban/23V-7567/2023 | . | S | . | . | . | V | . | . | . | . | . | . | . | . |  | . | . | . | . | . | . | . | T |
| A/Cameroon/1742/2023 | . | . | . | L | . | V | . | . | . | . | . | . | . | . | Y | . | . | . | . | . | . | . | T |
| A/Yaounde/23V-10944/2023 | . | . | . | . | . | V | . | . | . | . | . | . | . | . |  | . | . | . | . | . | W | . | T |
| A/Cameroon/9812/2023 | . | . | . | . | A | V | . | . | . | . | . | . | . | . |  | . | . | . | . | . | . | . | T |
| A/Cameroon/9092/2023 | . | . | . | . | . | V | . | . | . | . | . | . | . | . | Y | . | . | . | . | . | . | . | T |
| A/Cameroon/2254/2024 | . | . | . | . | . | V | . | . | . | . | . | . | . | . |  | . | . | . | T | . | . | . | T |
| A/Cameroon/2252/2024 | . | . | . | . | . | V | . | . | . | . | . | . | . | . |  | . | . | . | T | . | . | . | T |
| A/Cameroon/3172/2024 | . | . | . | . | . | V | . | . | . | . | . | . | . | . |  | . | . | . | T | . | . | . | T |
| A/Cameroon/1100/2024 | . | . | . | . | . | V | . | . | . | . | . | . | . | . |  | . | . | . | T | . | . | . | T |
| A/Yaounde/23V-10499/2023 | . | . | . | . | . | V | . | . | . | . | . | . | . | I |  | . | . | . | . | . | . | . | T |
| A/Cameroon/9072/2023 | . | . | . | V | . | V | A | . | . | . | . | . | . | . | Y | . | . | . | . | . | . | . | T |
| A/Cameroon/2919/2023 | . | . | . | . | . | V | . | . | N | . | . | . | R | . |  | . | . | . | . | . | . | . | T |
| A/Yaounde/23V-12684/2023 | - | . | . | . | . | V | . | . | . | . | . | . | . | . |  | . | . | . | . | . | . | . | T |
| A/Bamenda/23V-9661/2023 | F | . | . | . | . | V | . | . | . | P | . | . | . | . | Y | . | . | K | . | . | . | . | T |
| A/Cameroon/8474/2023 | F | . | L | . | . | V | . | . | . | P | . | . | . | . |  | . | . | . | . | . | . | . | T |
| A/Douala/23V-8444/2023 | F | . | S | . | . | V | . | . | . | P | . | . | . | . | Y | . | E | . | . | . | . | . | T |
| A/Cameroon/2500/2024 | . | . | . | . | . | . | . | . | . | . | . | H | . | . |  | . | . | . | . | K | . | . | T |
| A/Cameroon/3152/2024 | . | . | . | . | . | . | . | . | . | . | . | H | . | . | Y | . | . | . | . | K | . | . | T |
| A/Cameroon/6984/2024 | . | . | . | . | . | . | . | F | . | . | . | H | . | . |  | . | . | . | . | K | . | . | T |
| A/Cameroon/5947/2024 | . | . | . | . | . | . | . | . | . | . | . | H | . | . | Y | . | . | . | . | K | . | . | T |
| A/Cameroon/7196/2024 | . | . | . | . | . | . | . | . | . | . | I | H | . | . |  | V | . | . | . | K | . | . | T |
| A/Cameroon/7198/2024 | . | . | . | . | . | . | . | . | . | . | I | H | . | . |  | V | . | . | . | K | . | . | T |
| A/Cameroon/7167/2024 | . | . | . | . | . | . | . | . | . | . | I | H | . | . | Y | V | . | . | . | K | . | . | T |
| A/Cameroon/6580/2024 | . | . | . | . | . | . | . | . | . | . | I | H | . | . | Y | V | . | . | . | K | . | F | T |
| A/Cameroon/6591/2024 | . | . | . | . | . | . | . | . | . | . | I | H | . | . |  | . | . | . | . | K | . | . | T |
