# Supplementary material for: Evolving Dynamics of Whole-Genome Influenza A/H3N2 Viruses Isolated in Cameroon
Source: Adv Virol. 2025 Sep 19;2025:3668615. doi: 10.1155/av/3668615 (PMC12473741; doi:10.1155/av/3668615)
Supplement: Supporting Information 4 — Supporting Table S4: List of mutation differences in the NP gene between Cameroon 2023-2024 viruses and the A/Darwin/6/2021 vaccine strain. [file 3668615.f4.docx]

**Supplementary Table S4**: List of mutation differences in the NP gene between Cameroon 2023–2024 viruses and the A/Darwin/6/2021 vaccine strain

|  | NP | | | | | | | | | | | |
| --- | --- | --- | --- | --- | --- | --- | --- | --- | --- | --- | --- | --- |
| Virus Strain | **129** | **136** | **186** | **220** | **236** | **359** | **418** | **423** | **432** | **456** | **480** | **482** |
| A/Darwin/6/2021(H3N2) | A | M | I | D | R | S | I | S | N | V | D | S |
| A/Cameroon/2925/2023 | . | . | . | . | . | . | . | . | . | . | . | . |
| A/Yaounde/23V-10944/2023 | S | I | V | E | . | . | L | . | S | . | . | . |
| A/Yaounde/23V-11465/2023 | S | I | V | E | . | . | L | . | S | . | . | . |
| A/Yaounde/23V-10497/2023 | S | I | V | E | . | . | L | . | S | . | . | . |
| A/Cameroon/9092/2023 | S | I | V | E | . | . | L | . | S | . | . | . |
| A/Cameroon/9812/2023 | S | I | V | E | . | . | L | . | S | . | . | . |
| A/Cameroon/2254/2024 | S | I | V | E | . | . | L | . | S | . | . | . |
| A/Cameroon/2252/2024 | S | I | V | E | . | . | L | . | S | . | . | . |
| A/Cameroon/3172/2024 | S | I | V | E | . | . | L | . | S | . | . | . |
| A/Douala/23V-12328/2023 | S | I | V | E | . | . | L | . | S | . | . | . |
| A/Cameroon/1100/2024 | S | I | V | E | . | . | L | . | S | . | . | . |
| A/Yaounde/23V-12684/2023 | S | I | V | E | . | . | L | . | . | . | . | . |
| A/Yaounde/23V-10499/2023 | S | I | V | E | . | . | L | . | S | . | . | . |
| A/Cameroon/10509/2023 | . | I | V | E | . | . | L | . | . | . | . | N |
| A/Foumban/23V-7567/2023 | . | I | V | E | . | . | L | . | . | . | . | N |
| A/Cameroon/541/2023 | . | I | V | E | . | . | L | . | . | . | . | N |
| A/Yaounde/23V-9072/2023 | . | I | V | E | . | . | L | . | . | . | . | . |
| A/Cameroon/1742/2023 | . | I | V | E | . | . | L | . | . | . | . | . |
| A/Cameroon/2919/2023 | . | I | V | E | . | . | L | . | . | . | . | . |
| A/Bamenda/23V-9661/2023 | . | I | . | E | . | . | L | . | . | . | E | N |
| A/Cameroon/8474/2023 | . | I | . | E | . | . | L | . | . | . | E | N |
| A/Douala/23V-8444/2023 | . | I | . | E | . | . | L | P | . | . | E | N |
| A/Cameroon/5947/2024 | . | L | . | E | K | . | L | . | . | . | . | . |
| A/Cameroon/2500/2024 | . | L | . | E | K | . | L | . | . | . | . | . |
| A/Cameroon/6984/2024 | . | L | . | E | K | . | L | . | . | . | . | . |
| A/Cameroon/3152/2024 | . | L | . | E | K | . | L | . | . | . | . | . |
| A/Cameroon/7196/2024 | . | L | . | E | K | L | L | . | . | . | . | . |
| A/Cameroon/7198/2024 | . | L | . | E | K | L | L | . | . | . | . | . |
| A/Cameroon/7167/2024 | . | L | . | E | K | L | L | . | . | . | . | . |
| A/Cameroon/6580/2024 | . | L | . | E | K | L | L | . | . | . | . | . |
| A/Cameroon/6591/2024 | . | L | . | E | K | L | L | . | . | M | . | . |
