# Supplementary material for: Evolving Dynamics of Whole-Genome Influenza A/H3N2 Viruses Isolated in Cameroon
Source: Adv Virol. 2025 Sep 19;2025:3668615. doi: 10.1155/av/3668615 (PMC12473741; doi:10.1155/av/3668615)
Supplement: Supporting Information 5 — Supporting Table S5: List of mutation differences in the NS gene between Cameroon 2023-2024 viruses and the A/Darwin/6/2021 vaccine strain. [file 3668615.f5.docx]

**Supplementary Table S5**: List of mutation differences in the NS1 gene between Cameroon 2023–2024 viruses and the A/Darwin/6/2021 vaccine strain

| **Virus Strains** | | **NS1** | |  | |  | |  | |  | |  | |  |  |  |  |  |  |
| --- | --- | --- | --- | --- | --- | --- | --- | --- | --- | --- | --- | --- | --- | --- | --- | --- | --- | --- | --- |
|  | | 18 | | 26 | | 33 | | 59 | | 60 | | 65 | | 70 | 76 | 82 | 108 | 124 | 127 |
| **A/Darwin/6/2021(H3N2)** | | I | | K | | L | | H | | V | | V | | K | A | V | K | M | N |
| A/Yaounde/23V-9072/2023 | | V | | N | | . | | . | | A | | I | | . | . | . | . | . | S |
| A/Yaounde/23V-12684/2023 | | V | | N | | . | | . | | A | | . | | . | . | . | . | . | S |
| A/Yaounde/23V-11465/2023 | | V | | N | | . | | . | | A | | . | | . | . | . | . | . | S |
| A/Yaounde/23V-10944/2023 | | V | | N | | . | | . | | A | | . | | . | . | . | . | . | S |
| A/Yaounde/23V-10499/2023 | | V | | N | | . | | . | | A | | . | | . | . | . | . | . | S |
| A/Yaounde/23V-10497/2023 | | V | | N | | . | | . | | A | | . | | . | . | . | . | . | S |
| A/Foumban/23V-9812/2023 | | V | | N | | . | | . | | A | | I | | . | . | . | . | . | S |
| A/Foumban/23V-7567/2023 | | V | | N | | . | | . | | T | | I | | . | T | . | . | . | S |
| A/Douala/23V-8444/2023 | | . | | . | | . | | . | | . | | . | | . | . | . | N | . | . |
| A/Douala/23V-12328/2023 | | V | | N | | . | | . | | A | | . | | . | . | . | . | . | S |
| A/Cameroon/9092/2023 | | V | | T | | . | | . | | A | | . | | . | . | . | . | . | S |
| A/Cameroon/8474/2023 | | . | | . | | . | | . | | . | | . | | . | . | . | N | . | . |
| A/Cameroon/7198/2024 | | . | | N | | I | | . | | A | | . | | . | . | A | . | I | . |
| A/Cameroon/7196/2024 | | . | | N | | I | | . | | A | | . | | . | . | A | . | I | . |
| A/Cameroon/7167/2024 | | . | | N | | I | | . | | A | | . | | . | . | A | . | I | . |
| A/Cameroon/6984/2024 | | . | | N | | I | | . | | A | | . | | . | . | A | . | I | . |
| A/Cameroon/6591/2024 | | . | | N | | I | | N | | A | | . | | . | . | A | . | I | . |
| A/Cameroon/6580/2024 | | . | | N | | I | | . | | A | | . | | . | . | A | . | I | . |
| A/Cameroon/5947/2024 | | . | | N | | I | | . | | A | | . | | . | . | A | . | I | . |
| A/Cameroon/541/2023 | | V | | N | | . | | . | | A | | I | | . | . | . | . | . | S |
| A/Cameroon/5150/2024 | | . | | N | | I | | . | | A | | . | | . | . | A | . | I | . |
| A/Cameroon/3172/2024 | | V | | N | | . | | . | | A | | . | | . | . | . | . | . | S |
| A/Cameroon/3152/2024 | | . | | N | | I | | . | | A | | . | | . | . | A | . | I | . |
| A/Cameroon/2925/2023 | | . | | . | | . | | . | | . | | . | | E | . | . | . | . | . |
| A/Cameroon/2919/2023 | | V | | N | | . | | . | | A | | I | | . | . | . | . | . | S |
| A/Cameroon/2500/2024 | | . | | N | | I | | . | | A | | . | | . | . | A | . | I | . |
| A/Cameroon/2254/2024 | | V | | N | | . | | . | | A | | . | | . | . | . | . | . | S |
| A/Cameroon/2252/2024 | | V | | N | | . | | . | | A | | . | | . | . | . | . | . | S |
| A/Cameroon/1742/2023 | | V | | N | | . | | . | | A | | I | | . | . | . | . | . | S |
| A/Cameroon/1100/2024 | | V | | N | | . | | . | | A | | . | | . | . | . | . | . | S |
| A/Cameroon/10509/2023 | | V | | N | | . | | . | | A | | I | | . | T | . | . | . | S |
| A/Bamenda/23V-9661/2023 | | . | | . | | . | | . | | . | | . | | . | . | . | N | . | . |
| 139 | 152 | | 171 | | 196 | | 207 | | 220 | | 227 | |  |  |  |  |  |  |  |
| G | E | | I | | K | | H | | R | | R | |  |  |  |  |  |  |  |
| . | D | | . | | E | | N | | . | | . | |  |  |  |  |  |  |  |
| . | D | | . | | . | | N | | . | | G | |  |  |  |  |  |  |  |
| . | D | | . | | . | | N | | . | | G | |  |  |  |  |  |  |  |
| . | D | | . | | . | | N | | W | | G | |  |  |  |  |  |  |  |
| . | D | | . | | . | | N | | . | | G | |  |  |  |  |  |  |  |
| . | D | | . | | . | | N | | . | | G | |  |  |  |  |  |  |  |
| . | D | | . | | E | | N | | . | | . | |  |  |  |  |  |  |  |
| . | D | | . | | . | | N | | . | | . | |  |  |  |  |  |  |  |
| . | . | | . | | . | | . | | . | | . | |  |  |  |  |  |  |  |
| . | D | | . | | . | | N | | . | | G | |  |  |  |  |  |  |  |
| . | D | | . | | . | | N | | . | | G | |  |  |  |  |  |  |  |
| . | . | | . | | . | | . | | . | | . | |  |  |  |  |  |  |  |
| . | . | | . | | . | | N | | . | | . | |  |  |  |  |  |  |  |
| . | . | | . | | . | | N | | . | | . | |  |  |  |  |  |  |  |
| . | . | | . | | . | | N | | . | | . | |  |  |  |  |  |  |  |
| . | . | | V | | . | | N | | . | | . | |  |  |  |  |  |  |  |
| . | . | | . | | . | | N | | . | | . | |  |  |  |  |  |  |  |
| . | . | | . | | . | | N | | . | | . | |  |  |  |  |  |  |  |
| . | . | | V | | . | | N | | . | | . | |  |  |  |  |  |  |  |
| . | D | | . | | . | | N | | . | | . | |  |  |  |  |  |  |  |
| . | . | | V | | . | | N | | . | | . | |  |  |  |  |  |  |  |
| . | D | | . | | . | | N | | . | | G | |  |  |  |  |  |  |  |
| . | . | | V | | . | | N | | . | | . | |  |  |  |  |  |  |  |
| . | . | | . | | . | | . | | . | | . | |  |  |  |  |  |  |  |
| . | D | | . | | . | | N | | . | | . | |  |  |  |  |  |  |  |
| D | . | | V | | . | | N | | . | | . | |  |  |  |  |  |  |  |
| . | D | | . | | . | | N | | . | | G | |  |  |  |  |  |  |  |
| . | D | | . | | . | | N | | . | | G | |  |  |  |  |  |  |  |
| . | D | | . | | . | | N | | . | | . | |  |  |  |  |  |  |  |
| . | D | | . | | . | | N | | . | | G | |  |  |  |  |  |  |  |
| . | D | | . | | . | | N | | . | | . | |  |  |  |  |  |  |  |
| . | . | | . | | . | | . | | . | | . | |  |  |  |  |  |  |  |

**Supplementary Table S5**: List of mutation differences in the NS2 gene between Cameroon 2023–2024 viruses and the A/Darwin/6/2021 vaccine strain

| **Virus Strains** | **NS2** |  |
| --- | --- | --- |
|  | 14 | 88 |
| **(A/Darwin/6/2021(H3N2)** | L | R |
| A/Yaounde/23V-9072/2023 | . | . |
| A/Yaounde/23V-12684/2023 | . | . |
| A/Yaounde/23V-11465/2023 | . | . |
| A/Yaounde/23V-10944/2023 | . | . |
| A/Yaounde/23V-10499/2023 | . | . |
| A/Yaounde/23V-10497/2023 | S | . |
| A/Foumban/23V-9812/2023 | . | . |
| A/Foumban/23V-7567/2023 | . | . |
| A/Douala/23V-8444/2023 | . | . |
| A/Douala/23V-12328/2023 | . | . |
| A/Cameroon/9092/2023 | . | . |
| A/Cameroon/8474/2023 | . | . |
| A/Cameroon/7198/2024 | . | K |
| A/Cameroon/7196/2024 | . | K |
| A/Cameroon/7167/2024 | . | K |
| A/Cameroon/6984/2024 | . | K |
| A/Cameroon/6591/2024 | . | K |
| A/Cameroon/6580/2024 | . | K |
| A/Cameroon/5947/2024 | . | K |
| A/Cameroon/541/2023 | . | . |
| A/Cameroon/5150/2024 | . | K |
| A/Cameroon/3172/2024 | . | . |
| A/Cameroon/3152/2024 | . | K |
| A/Cameroon/2925/2023 | . | . |
| A/Cameroon/2919/2023 | . | . |
| A/Cameroon/2500/2024 | . | K |
| A/Cameroon/2254/2024 | . | . |
| A/Cameroon/2252/2024 | . | . |
| A/Cameroon/1742/2023 | . | . |
| A/Cameroon/1100/2024 | . | . |
| A/Cameroon/10509/2023 | . | . |
| A/Bamenda/23V-9661/2023 | . | . |
