# Supplementary material for: Evolving Dynamics of Whole-Genome Influenza A/H3N2 Viruses Isolated in Cameroon
Source: Adv Virol. 2025 Sep 19;2025:3668615. doi: 10.1155/av/3668615 (PMC12473741; doi:10.1155/av/3668615)
Supplement: Supporting Information 6 — Supporting Table S6: List of mutation differences in the PA gene between Cameroon 2023-2024 viruses and the A/Darwin/6/2021 vaccine strain. [file 3668615.f6.docx]

**Supplementary Table S6**: List of mutation differences in the PA gene between Cameroon 2023–2024 viruses and the A/Darwin/6/2021 vaccine strain

| **Virus Strains** | **PA** |  |  |  |  |  |  |  |  |  |  |  |  |
| --- | --- | --- | --- | --- | --- | --- | --- | --- | --- | --- | --- | --- | --- |
|  | 20 | 90 | 99 | 100 | 101 | 142 | 158 | 186 | 213 | 269 | 277 | 311 | 321 |
| **A/Darwin/6/2021(H3N2** | A | V | E | A | G | N | R | G | R | K | Y | I | Y |
| A/Cameroon/2925/2023 | . | . | G | . | . | . | . | . | . | . | . | . | C |
| A/Cameroon/2254/2024 | T | . | G | . | . | . | K | . | K | R | H | . | C |
| A/Cameroon/2252/2024 | T | . | G | . | . | . | K | . | K | R | H | . | C |
| A/Cameroon/3172/2024 | T | . | G | . | . | . | K | . | K | R | H | . | C |
| A/Cameroon/1100/2024 | T | . | G | . | . | . | K | . | K | R | H | . | C |
| A/Yaounde/23V-10497/2023 | T | . | G | . | . | . | K | . | K | R | H | . | C |
| A/Cameroon/9812/2023 | T | . | G | . | . | . | K | . | K | R | H | . | C |
| A/Yaounde/23V-12684/2023 | T | I | G | . | . | . | K | . | K | R | H | . | C |
| A/Cameroon/9092/2023 | T | . | G | . | . | . | K | . | K | R | H | . | C |
| A/Yaounde/23V-9072/2023 | T | . | G | . | . | . | . | . | . | R | H | . | C |
| A/Cameroon/541/2023 | T | . | G | . | . | . | . | . | . | R | H | . | C |
| A/Cameroon/1742/2023 | T | . | G | . | . | . | . | . | . | R | H | . | C |
| A/Cameroon/2919/2023 | T | . | G | . | . | . | . | . | . | R | H | . | C |
| A/Foumban/23V-7567/2023 | T | . | G | . | . | . | . | . | . | R | H | . | C |
| A/Cameroon/3152/2024 | . | . | G | . | E | K | . | . | . | . | . | M | C |
| A/Cameroon/2500/2024 | . | . | G | . | E | K | . | . | . | . | . | M | C |
| A/Cameroon/7196/2024 | . | . | G | . | E | K | . | . | . | . | . | M | C |
| A/Cameroon/6580/2024 | . | . | G | . | E | K | . | . | . | . | . | M | C |
| A/Cameroon/7167/2024 | . | . | G | . | E | K | . | . | . | . | . | M | C |
| A/Cameroon/6591/2024 | . | . | G | . | E | K | . | . | . | . | . | M | C |
| A/Cameroon/7198/2024 | . | . | G | . | E | K | . | . | . | . | . | M | C |
| A/Cameroon/6580/2024 | . | . | G | . | E | K | . | . | . | . | . | M | C |
| A/Cameroon/5947/2024 | . | . | G | . | E | K | . | . | . | . | . | M | C |
| A/Cameroon/6984/2024 | . | . | G | . | E | K | . | S | . | . | . | M | C |
| A/Bamenda/23V-9661/2023 | . | . | G | V | . | . | . | . | . | . | . | M | C |
| A/Cameroon/8474/2023 | . | . | G | . | . | . | . | . | . | . | . | M | C |
| 325 | 327 | 343 | 374 | 400 | 402 | 407 | 497 | 551 | 588 | 592 | 605 | 660 | 684 |
| P | E | S | M | L | A | V | K | R | S | I | K | S | G |
| . | . | . | . | . | T | I | . | . | . | . | . | A | . |
| . | . | . | . | I | S | I | R | . | . | . | . | A | . |
| . | . | . | . | I | S | I | R | . | . | . | . | A | . |
| . | . | . | . | I | S | I | R | . | . | . | . | A | . |
| . | . | . | . | I | S | I | R | . | . | . | . | A | . |
| S | . | . | . | I | S | I | R | . | . | . | . | A | . |
| . | . | . | . | I | S | I | R | . | . | . | . | A | . |
| . | . | . | I | I | S | I | R | K | . | . | . | A | . |
| . | K | . | . | I | S | I | R | . | . | . | . | A | . |
| . | . | A | . | . | S | I | R | . | . | V | . | A | R |
| . | . | . | . | . | S | I | R | . | . | . | . | A | R |
| . | . | . | . | . | S | I | R | . | . | . | . | A | R |
| . | . | . | . | . | S | I | R | . | P | . | . | A | R |
| . | . | . | . | . | S | I | R | . | . | . | . | A | R |
| . | . | . | . | . | S | I | ? | . | . | . | R | A | . |
| . | . | . | . | . | S | I | . | . | . | . | R | A | . |
| . | . | . | . | . | S | I | . | . | . | . | R | A | . |
| . | . | . | . | . | S | I | . | . | . | . | R | A | . |
| . | . | . | . | . | S | I | . | . | . | . | R | A | . |
| . | . | . | . | . | S | I | . | . | . | . | R | A | . |
| . | . | . | . | . | S | I | . | . | . | . | R | A | . |
| . | . | . | . | . | S | I | . | . | . | . | R | A | . |
| . | . | . | . | . | S | I | . | . | . | . | R | A | . |
| . | . | . | . | . | S | I | . | . | . | . | R | A | . |
| S | V | . | . | . | S | I | . | . | . | . | R | A | . |
| S | V | . | . | . | S | I | . | . | . | . | R | A | . |
