# Supplementary material for: Evolving Dynamics of Whole-Genome Influenza A/H3N2 Viruses Isolated in Cameroon
Source: Adv Virol. 2025 Sep 19;2025:3668615. doi: 10.1155/av/3668615 (PMC12473741; doi:10.1155/av/3668615)
Supplement: Supporting Information 7 — Supporting Table S7: List of mutation differences in the PB1 gene between Cameroon 2023-2024 viruses and the A/Darwin/6/2021 vaccine strain. [file 3668615.f7.docx]

**Supplementary Table S7**: List of mutation differences in the PB1 gene between Cameroon 2023–2024 viruses and the A/Darwin/6/2021 vaccine strain

| **Virus Strain** | | **PB1** | |  | |  | |  |  |  |  |  |  |  |
| --- | --- | --- | --- | --- | --- | --- | --- | --- | --- | --- | --- | --- | --- | --- |
|  | | 10 | | 64 | | 122 | | 149 | 178 | 194 | 200 | 375 | 386 | 502 |
| **(A/Darwin/6/2021(H3N2))** | | L | | P | | L | | V | E | N | V | N | R | V |
| A/Cameroon/2925/2023 | | . | | . | | . | | . | . | . | . | S | . | . |
| A/Cameroon/541/2023 | | . | | . | | . | | . | . | . | . | S | . | . |
| A/Cameroon/1742/2023 | | . | | . | | . | | . | K | . | . | S | . | . |
| A/Foumban/23V-7567/2023 | | . | | . | | . | | . | . | . | . | S | . | . |
| A/Cameroon/2919/2023 | | . | | . | | . | | . | . | . | . | S | . | . |
| A/Cameroon/2252/2024 | | . | | . | | . | | . | . | . | I | S | . | . |
| A/Cameroon/2254/2024 | | . | | . | | . | | . | . | . | I | S | . | . |
| A/Cameroon/1100/2024 | | . | | . | | . | | . | . | . | I | S | . | . |
| A/Cameroon/3172/2024 | | . | | . | | . | | . | . | . | I | S | . | . |
| A/Yaounde/23V-10497/2023 | | . | | S | | . | | . | . | . | I | S | . | . |
| A/Cameroon/9812/2023 | | . | | . | | . | | . | . | . | I | S | . | . |
| A/Cameroon/9092/2023 | | . | | . | | . | | . | . | . | I | S | . | . |
| A/Yaounde/23V-12684/2023 | | . | | . | | P | | . | . | . | I | S | . | . |
| A/Cameroon/2500/2024 | | . | | . | | . | | . | . | . | . | S | K | . |
| A/Cameroon/6984/2024 | | ? | | . | | . | | . | . | . | . | S | K | R |
| A/Cameroon/5947/2024 | | . | | . | | . | | I | . | S | . | S | K | . |
| A/Cameroon/7196/2024 | | . | | . | | . | | . | . | . | . | S | . | . |
| A/Cameroon/7198/2024 | | . | | . | | . | | . | . | . | . | S | . | . |
| A/Cameroon/6580/2024 | | . | | . | | . | | . | . | . | . | S | . | . |
| A/Cameroon/6591/2024 | | . | | . | | . | | . | . | . | . | S | . | . |
| A/Cameroon/7167/2024 | | I | | . | | . | | . | . | . | . | S | . | . |
| 527 | 565 | | 597 | | 758 | |  |  |  |  |  |  |  |  |
| V | D | | N | | * | |  |  |  |  |  |  |  |  |
| . | . | | . | | ? | |  |  |  |  |  |  |  |  |
| . | . | | . | | ? | |  |  |  |  |  |  |  |  |
| . | . | | . | | ? | |  |  |  |  |  |  |  |  |
| . | . | | . | | Q | |  |  |  |  |  |  |  |  |
| . | . | | . | | ? | |  |  |  |  |  |  |  |  |
| I | . | | . | | Q | |  |  |  |  |  |  |  |  |
| I | . | | . | | Q | |  |  |  |  |  |  |  |  |
| I | . | | . | | ? | |  |  |  |  |  |  |  |  |
| I | . | | . | | Q | |  |  |  |  |  |  |  |  |
| . | . | | . | | Q | |  |  |  |  |  |  |  |  |
| . | . | | . | | Q | |  |  |  |  |  |  |  |  |
| . | . | | . | | Q | |  |  |  |  |  |  |  |  |
| . | N | | S | | Q | |  |  |  |  |  |  |  |  |
| . | . | | . | | ? | |  |  |  |  |  |  |  |  |
| . | . | | . | | Q | |  |  |  |  |  |  |  |  |
| . | . | | . | | Q | |  |  |  |  |  |  |  |  |
| . | . | | . | | ? | |  |  |  |  |  |  |  |  |
| . | . | | . | | ? | |  |  |  |  |  |  |  |  |
| . | . | | . | | Q | |  |  |  |  |  |  |  |  |
| . | . | | . | | Q | |  |  |  |  |  |  |  |  |
| . | . | | . | | ? | |  |  |  |  |  |  |  |  |
