# Supplementary material for: Evolving Dynamics of Whole-Genome Influenza A/H3N2 Viruses Isolated in Cameroon
Source: Adv Virol. 2025 Sep 19;2025:3668615. doi: 10.1155/av/3668615 (PMC12473741; doi:10.1155/av/3668615)
Supplement: Supporting Information 8 — Supporting Table S8: List of mutation differences in the PB2 gene between Cameroon 2023-2024 viruses and the A/Darwin/6/2021 vaccine strain. [file 3668615.f8.docx]

**Supplementary Table S8**: List of mutation differences in the PB2 gene between Cameroon 2023–2024 viruses and the A/Darwin/6/2021 vaccine strain

| **Virus Strain** | | **PB2** |  | |  | |  | |  | |  | |  | |  |  |  | |  | |  | |  | |  |
| --- | --- | --- | --- | --- | --- | --- | --- | --- | --- | --- | --- | --- | --- | --- | --- | --- | --- | --- | --- | --- | --- | --- | --- | --- | --- |
|  | | 6 | 58 | | 59 | | 63 | | 87 | | 88 | | 89 | | 98 | 99 | 105 | | 107 | | 114 | | 127 | |  |
| **A/Darwin/6/2021(H3N2)** | | E | T | | A | | I | | D | | R | | V | | W | W | V | | D | | V | | H | |  |
| A/Cameroon/2252/2024 | | . | . | | . | | . | | . | | . | | . | | . | . | . | | . | | . | | . | |  |
| A/Cameroon/1100/2024 | | G | . | | . | | . | | . | | . | | . | | . | . | . | | . | | . | | . | |  |
| A/Cameroon/2254/2024 | | . | . | | . | | . | | . | | . | | . | | . | . | . | | . | | . | | . | |  |
| A/Cameroon/9092/2023 | | . | . | | . | | . | | . | | . | | . | | . | . | . | | . | | . | | . | |  |
| A/Cameroon/3172/2024 | | . | . | | . | | . | | . | | . | | . | | . | . | . | | . | | . | | . | |  |
| A/Yaounde/23V-10497/2023 | | . | . | | . | | . | | . | | . | | . | | . | . | . | | . | | . | | . | |  |
| A/Cameroon/9812/2023 | | . | . | | . | | . | | . | | . | | . | | . | . | . | | . | | . | | . | |  |
| A/Yaounde/23V-12684/2023 | | . | . | | . | | . | | . | | . | | . | | . | . | . | | . | | . | | . | |  |
| A/Cameroon/9072/2023 | | . | . | | . | | . | | . | | . | | . | | . | . | . | | . | | . | | . | |  |
| A/Cameroon/8474/2023 | | . | . | | . | | . | | N | | . | | . | | . | . | . | | . | | . | | . | |  |
| A/Cameroon/541/2023 | | . | . | | . | | L | | . | | . | | . | | . | . | . | | . | | . | | . | |  |
| A/Foumban/23V-7567/2023 | | . | . | | . | | . | | . | | . | | . | | . | . | . | | . | | . | | . | |  |
| A/Cameroon/1742/2023 | | . | . | | . | | . | | . | | . | | . | | . | . | . | | . | | . | | . | |  |
| A/Cameroon/2919/2023 | | . | . | | . | | . | | . | | . | | . | | . | . | . | | . | | . | | . | |  |
| A/Cameroon/2925/2023 | | . | . | | . | | . | | . | | . | | . | | . | . | . | | . | | . | | . | |  |
| A/Cameroon/2500/2024 | | . | . | | . | | . | | . | | . | | . | | . | . | . | | N | | . | | . | |  |
| A/Cameroon/5947/2024 | | . | . | | . | | . | | . | | . | | . | | . | . | . | | N | | . | | . | |  |
| A/Cameroon/3152/2024 | | . | . | | . | | . | | . | | . | | . | | . | . | . | | N | | . | | . | |  |
| A/Cameroon/6984/2024 | | . | . | | . | | . | | . | | . | | . | | . | . | . | | N | | . | | . | |  |
| A/Cameroon/7196/2024 | | . | . | | . | | . | | . | | . | | . | | . | . | . | | N | | . | | . | |  |
| A/Cameroon/7198/2024 | | . | . | | . | | . | | . | | . | | . | | . | . | . | | N | | . | | . | |  |
| A/Cameroon/6591/2024 | | . | . | | . | | . | | . | | . | | . | | . | . | . | | N | | . | | . | |  |
| A/Cameroon/7167/2024 | | . | . | | . | | . | | . | | . | | . | | . | . | . | | N | | . | | . | |  |
| A/Cameroon/6580/2024 | | . | . | | . | | . | | . | | . | | . | | . | . | . | | N | | . | | . | |  |
| A/Bamenda/23V-9661/2023 | | . | N | | D | | . | | N | | G | | L | | C | G | M | | . | | M | | Q | |  |
| 140 | 147 | 154 | | 188 | | 189 | | 194 | | 210 | | 237 | | 243 | | 244 | | 251 | | 262 | | 275 | |  |  |
| K | I | L | | E | | K | | R | | E | | G | | M | | Y | | R | | A | | D | |  |  |
| . | . | . | | . | | . | | . | | . | | . | | . | | . | | . | | . | | . | |  |  |
| . | . | . | | . | | . | | . | | . | | . | | . | | . | | . | | . | | . | |  |  |
| . | . | . | | . | | . | | . | | . | | . | | . | | . | | . | | . | | . | |  |  |
| . | . | . | | . | | . | | . | | . | | . | | . | | . | | . | | . | | . | |  |  |
| . | . | . | | . | | . | | . | | . | | . | | . | | . | | . | | . | | . | |  |  |
| . | . | . | | . | | . | | . | | . | | . | | . | | . | | . | | . | | . | |  |  |
| . | . | . | | . | | . | | . | | . | | . | | . | | . | | . | | . | | . | |  |  |
| . | . | . | | . | | . | | . | | . | | . | | . | | . | | . | | . | | . | |  |  |
| . | . | . | | . | | . | | . | | . | | . | | . | | . | | . | | . | | . | |  |  |
| . | . | . | | . | | . | | . | | . | | . | | . | | . | | . | | . | | . | |  |  |
| . | . | . | | . | | . | | . | | . | | . | | . | | . | | . | | . | | . | |  |  |
| . | . | . | | . | | . | | . | | . | | . | | . | | . | | . | | . | | . | |  |  |
| . | . | . | | . | | . | | . | | . | | . | | . | | . | | . | | . | | . | |  |  |
| . | . | . | | . | | . | | . | | . | | . | | . | | . | | . | | . | | . | |  |  |
| . | . | . | | . | | . | | . | | . | | . | | . | | . | | K | | . | | . | |  |  |
| . | T | . | | . | | . | | . | | . | | . | | . | | . | | . | | . | | . | |  |  |
| . | T | . | | . | | . | | . | | . | | . | | . | | . | | . | | . | | . | |  |  |
| . | T | . | | . | | . | | . | | . | | . | | . | | . | | . | | . | | . | |  |  |
| . | T | . | | . | | . | | . | | . | | . | | . | | . | | . | | . | | . | |  |  |
| . | T | . | | . | | . | | . | | . | | . | | . | | . | | . | | . | | . | |  |  |
| . | T | . | | . | | . | | . | | . | | . | | . | | . | | . | | . | | . | |  |  |
| . | T | . | | . | | . | | . | | . | | . | | . | | . | | . | | . | | . | |  |  |
| . | T | . | | . | | . | | . | | . | | . | | . | | . | | . | | . | | . | |  |  |
| . | T | . | | . | | . | | . | | . | | . | | . | | . | | . | | . | | . | |  |  |
| N | . | P | | D | | E | | Q | | N | | R | | I | | C | | . | | V | | N | |  |  |
| 278 | 282 | 320 | | 334 | | 340 | | 341 | | 344 | | 368 | | 369 | | 374 | | 384 | | 386 | | 405 | | 410 | |
| A | E | S | | S | | R | | E | | V | | K | | R | | L | | L | | V | | S | | V | |
| . | . | . | | . | | K | | . | | . | | . | | . | | . | | . | | . | | . | | . | |
| . | . | . | | . | | K | | . | | . | | . | | . | | . | | . | | . | | . | | . | |
| . | . | . | | . | | K | | . | | . | | . | | . | | . | | . | | . | | . | | . | |
| . | . | . | | . | | ? | | . | | . | | . | | . | | . | | . | | . | | . | | . | |
| . | . | . | | . | | K | | . | | . | | . | | . | | . | | . | | . | | . | | . | |
| . | . | . | | . | | K | | . | | . | | . | | . | | . | | . | | . | | . | | . | |
| . | . | . | | . | | K | | . | | . | | . | | . | | . | | . | | . | | . | | . | |
| . | . | . | | . | | K | | . | | . | | . | | . | | . | | . | | . | | . | | . | |
| . | . | . | | . | | K | | . | | . | | . | | . | | . | | . | | . | | . | | . | |
| . | . | . | | . | | K | | . | | . | | . | | . | | . | | . | | . | | . | | . | |
| . | . | . | | . | | K | | . | | . | | . | | . | | . | | . | | . | | . | | . | |
| . | . | . | | . | | K | | . | | . | | . | | . | | . | | . | | . | | . | | . | |
| . | . | . | | . | | K | | . | | . | | . | | . | | . | | . | | . | | . | | . | |
| . | . | . | | . | | K | | . | | . | | . | | . | | . | | . | | . | | . | | . | |
| . | . | . | | . | | . | | . | | . | | . | | . | | . | | . | | . | | . | | . | |
| . | . | . | | . | | . | | . | | . | | . | | . | | . | | . | | . | | . | | M | |
| . | . | . | | . | | . | | . | | . | | . | | . | | . | | . | | . | | . | | M | |
| . | . | . | | . | | . | | . | | . | | . | | . | | . | | . | | . | | . | | M | |
| . | . | . | | . | | . | | . | | . | | . | | . | | . | | . | | . | | . | | M | |
| . | . | . | | . | | . | | . | | . | | . | | . | | . | | . | | . | | . | | M | |
| . | . | . | | . | | . | | . | | . | | . | | . | | . | | . | | . | | . | | M | |
| . | . | . | | . | | . | | . | | . | | . | | . | | . | | . | | . | | . | | M | |
| . | . | . | | . | | . | | . | | . | | . | | . | | . | | F | | . | | . | | M | |
| . | . | . | | . | | . | | . | | . | | . | | . | | . | | F | | . | | . | | M | |
| P | K | C | | A | | K | | G | | A | | R | | K | | R | | . | | E | | P | | . | |
| 411 | 421 | 433 | | 436 | | 448 | | 461 | | 497 | | 527 | | 541 | | 554 | | 563 | | 569 | | 596 | | 599 | |
| I | V | Q | | R | | N | | V | | S | | L | | G | | I | | Q | | A | | V | | L | |
| . | . | . | | . | | . | | . | | . | | . | | . | | . | | . | | . | | . | | . | |
| . | . | . | | . | | . | | . | | . | | . | | . | | . | | . | | . | | . | | . | |
| . | . | . | | . | | . | | . | | . | | . | | . | | . | | . | | . | | . | | . | |
| . | . | . | | . | | . | | . | | . | | . | | . | | . | | . | | . | | . | | . | |
| . | . | . | | . | | . | | . | | . | | . | | . | | . | | . | | . | | . | | . | |
| . | . | . | | . | | . | | I | | . | | . | | . | | . | | . | | . | | . | | . | |
| . | . | . | | . | | . | | . | | . | | . | | . | | . | | . | | . | | . | | . | |
| . | . | . | | . | | . | | I | | . | | . | | . | | V | | . | | . | | . | | . | |
| . | . | . | | . | | . | | . | | . | | . | | . | | . | | . | | . | | . | | . | |
| . | . | . | | . | | . | | . | | . | | . | | . | | . | | . | | . | | . | | . | |
| . | . | . | | . | | . | | . | | . | | . | | . | | . | | . | | . | | . | | . | |
| . | . | . | | . | | . | | . | | . | | . | | . | | . | | . | | . | | . | | . | |
| . | . | . | | . | | . | | . | | . | | . | | . | | . | | . | | . | | . | | . | |
| . | . | . | | . | | . | | . | | . | | . | | . | | . | | . | | . | | . | | . | |
| . | . | . | | . | | . | | . | | . | | . | | . | | . | | . | | . | | . | | . | |
| . | . | . | | . | | . | | . | | . | | . | | . | | . | | . | | . | | . | | . | |
| . | . | . | | . | | . | | . | | . | | . | | . | | . | | . | | . | | . | | . | |
| . | . | . | | . | | . | | . | | . | | . | | . | | . | | . | | . | | . | | . | |
| . | . | . | | . | | . | | . | | . | | . | | . | | . | | . | | . | | . | | . | |
| . | . | . | | . | | . | | . | | . | | . | | . | | . | | . | | . | | . | | . | |
| . | . | . | | . | | . | | . | | . | | . | | . | | . | | . | | . | | . | | . | |
| . | . | . | | . | | . | | . | | . | | . | | . | | . | | . | | . | | . | | . | |
| . | . | . | | . | | . | | . | | . | | . | | . | | . | | . | | T | | . | | . | |
| . | . | . | | . | | . | | . | | . | | . | | . | | . | | . | | . | | . | | . | |
| V | I | P | | G | | D | | . | | M | | V | | S | | . | | R | | T | | F | | P | |
| 605 | 608 | 650 | | 676 | | 682 | |  |  |  |  |  |  |  |  |  |  |  |  |  |  |  |  |  |  |
| D | G | R | | I | | S | |  |  |  |  |  |  |  |  |  |  |  |  |  |  |  |  |  |  |
| . | . | . | | . | | . | |  |  |  |  |  |  |  |  |  |  |  |  |  |  |  |  |  |  |
| . | . | . | | . | | . | |  |  |  |  |  |  |  |  |  |  |  |  |  |  |  |  |  |  |
| . | . | . | | . | | . | |  |  |  |  |  |  |  |  |  |  |  |  |  |  |  |  |  |  |
| . | . | . | | . | | . | |  |  |  |  |  |  |  |  |  |  |  |  |  |  |  |  |  |  |
| . | . | . | | . | | . | |  |  |  |  |  |  |  |  |  |  |  |  |  |  |  |  |  |  |
| . | . | . | | . | | . | |  |  |  |  |  |  |  |  |  |  |  |  |  |  |  |  |  |  |
| . | . | . | | . | | . | |  |  |  |  |  |  |  |  |  |  |  |  |  |  |  |  |  |  |
| . | . | . | | M | | . | |  |  |  |  |  |  |  |  |  |  |  |  |  |  |  |  |  |  |
| . | . | . | | . | | N | |  |  |  |  |  |  |  |  |  |  |  |  |  |  |  |  |  |  |
| . | . | . | | . | | . | |  |  |  |  |  |  |  |  |  |  |  |  |  |  |  |  |  |  |
| . | . | . | | . | | . | |  |  |  |  |  |  |  |  |  |  |  |  |  |  |  |  |  |  |
| . | . | . | | . | | . | |  |  |  |  |  |  |  |  |  |  |  |  |  |  |  |  |  |  |
| . | . | . | | . | | . | |  |  |  |  |  |  |  |  |  |  |  |  |  |  |  |  |  |  |
| . | . | . | | . | | . | |  |  |  |  |  |  |  |  |  |  |  |  |  |  |  |  |  |  |
| . | . | . | | . | | . | |  |  |  |  |  |  |  |  |  |  |  |  |  |  |  |  |  |  |
| . | . | . | | . | | . | |  |  |  |  |  |  |  |  |  |  |  |  |  |  |  |  |  |  |
| . | . | . | | . | | . | |  |  |  |  |  |  |  |  |  |  |  |  |  |  |  |  |  |  |
| . | . | . | | . | | . | |  |  |  |  |  |  |  |  |  |  |  |  |  |  |  |  |  |  |
| . | . | . | | . | | . | |  |  |  |  |  |  |  |  |  |  |  |  |  |  |  |  |  |  |
| . | . | . | | . | | . | |  |  |  |  |  |  |  |  |  |  |  |  |  |  |  |  |  |  |
| . | . | . | | . | | . | |  |  |  |  |  |  |  |  |  |  |  |  |  |  |  |  |  |  |
| . | . | . | | . | | . | |  |  |  |  |  |  |  |  |  |  |  |  |  |  |  |  |  |  |
| . | . | . | | . | | . | |  |  |  |  |  |  |  |  |  |  |  |  |  |  |  |  |  |  |
| . | . | . | | . | | . | |  |  |  |  |  |  |  |  |  |  |  |  |  |  |  |  |  |  |
| N | R | W | | . | | . | |  |  |  |  |  |  |  |  |  |  |  |  |  |  |  |  |  |  |
