# Supplementary material for: Evolving Dynamics of Whole-Genome Influenza A/H3N2 Viruses Isolated in Cameroon
Source: Adv Virol. 2025 Sep 19;2025:3668615. doi: 10.1155/av/3668615 (PMC12473741; doi:10.1155/av/3668615)
Supplement: Supporting Information 9 — Supporting Table S9: List of mutation differences in the MP gene between Cameroon 2023-2024 viruses and the A/Darwin/9/2021 vaccine strain. [file 3668615.f9.docx]

**Supplementary Table S9**: List of mutation differences in the M gene between Cameroon 2023–2024 viruses and the A/Darwin/6/2021 vaccine strain

| **Virus strain** | **M1** |
| --- | --- |
|  | 85 |
| **A/Darwin/6/2021(H3N2))** | N |
| A/Cameroon/2925/2023 | . |
| A/Douala/23V-10940/2023 | . |
| A/Douala/23V-12328/2023 | . |
| A/Cameroon/2254/2024 | . |
| A/Cameroon/2252/2024 | . |
| A/Yaounde/23V-10497/2023 | S |
| A/Cameroon/9092/2023 | . |
| A/Cameroon/3172/2024 | . |
| A/Cameroon/1100/2024 | S |
| A/Foumban/23V-9812/2023 | . |
| A/Yaounde/23V-9072/2023 | . |
| A/Cameroon/10509/2023 | . |
| A/Foumban/23V-7567/2023 | S |
| A/Cameroon/541/2023 | . |
| A/Cameroon/1742/2023 | . |
| A/Cameroon/2919/2023 | . |
| A/Yaounde/23V-10944/2023 | . |
| A/Yaounde/23V-11465/2023 | . |
| A/Yaounde/23V-12684/2023 | . |
| A/Yaounde/23V-10499/2023 | . |
| A/Bamenda/23V-9661/2023 | . |
| A/Cameroon/8474/2023 | . |
| A/Douala/23V-8444/2023 | . |
| A/Cameroon/5150/2024 | . |
| A/Cameroon/6984/2024 | . |
| A/Cameroon/2500/2024 | . |
| A/Cameroon/5947/2024 | . |
| A/Cameroon/3152/2024 | . |
| A/Cameroon/7196/2024 | . |
| A/Cameroon/7198/2024 | . |

| **Virus Strain** | **M2** |  |  |  |  |  |  |  |  |  |
| --- | --- | --- | --- | --- | --- | --- | --- | --- | --- | --- |
|  | 24 | 25 | 27 | 31 | 52 | 54 | 59 | 66 | 82 | 85 |
| **(A/Darwin/6/2021(H3N2))** | F | L | V | S | Y | F | L | E | S | N |
| A/Cameroon/2925/2023 | . | . | . | . | . | . | . |  | . | . |
| A/Cameroon/541/2023 | . | . | . | . | . | . | . |  | . | . |
| A/Cameroon/1742/2023 | . | . | . | . | K | . | . |  | . | . |
| A/Foumban/23V-7567/2023 | . | . | . | . | . | . | . |  | . | S |
| A/Cameroon/2919/2023 | . | . | . | . | . | . | . |  | . | . |
| A/Cameroon/2252/2024 | . | . | . | . | . | . |  |  | . | . |
| A/Cameroon/2254/2024 | . | . | . | . | . | . |  |  | . | . |
| A/Cameroon/1100/2024 | . | . | . | . | . | . |  | K | . | S |
| A/Cameroon/3152/2024 | L | P | . | . | . | L | I |  | . | . |
| A/Yaounde/23V-10497/2023 | . | S | I | N | . | . | I |  | N | . |
| A/Cameroon/9812/2023 | . | . | . | . | . | . | I |  | . | . |
| A/Cameroon/9092/2023 | . | . | . | . | . | . | I |  | . | . |
| A/Yaounde/23V-12684/2023 | . | . | P | . | . | . | I |  | . | . |
| A/Cameroon/2500/2024 | . | . | . | . | . | . | . |  | K | . |
| A/Cameroon/6984/2024 |  | . | . | . | . | . | . |  | K | R |
| A/Cameroon/5947/2024 | . | . | . | I | . | S | . |  | K | . |
| A/Cameroon/7196/2024 | . | . | . | . | . | . | . |  | . | . |
| A/Cameroon/7198/2024 | . | . | . | . | . | . | . |  | . | . |
| A/Cameroon/6580/2024 | . | . | . | . | . | . | . |  | . | . |
| A/Cameroon/6591/2024 | . | . | . | . | . | . | . |  | . | . |
| A/Cameroon/7167/2024 | I | . | . | . | . | . | . |  | . | . |
